# Efficacy and safety of anisodamine as adjuvant therapy in septic shock: a systematic review and meta-analysis

**DOI:** 10.3389/fphar.2026.1976742

**Published:** 2026-10-30

**Authors:** Yang Chen, Yuxuan Feng, Guangyao Zhong, Wei Wang, Haixia Zhou, Shujuan Yuan, Liqing Guan, Wei He, Zhenjie Tan, Weirong Yin

**Affiliations:** 1 Department of Clinical Pharmacy, The Sixth Affiliated Hospital of Jinan University, Dongguan, China; 2 School of Southern Medicine, Guangdong Jiangmen Chinese Medicine College, Jiangmen, China; 3 Department of Intensive Care Unit, The Sixth Affiliated Hospital of Jinan University, Dongguan, China; 4 Department of Pharmacy, Zhujiang Hospital, Southern Medical University, Guangzhou, China

**Keywords:** anisodamine, meta-analysis, microcirculation, septic shock, tissue perfusion

## Abstract

**Background:**

Some patients with septic shock still exhibit microcirculatory dysfunction and impaired tissue oxygen metabolism (macro–micro dissociation), even when macrohemodynamic targets are achieved. Anisodamine may improve patients’ symptoms through multiple mechanisms, including anti-inflammatory and immunomodulatory effects mediated by the cholinergic anti-inflammatory pathway and the relief of microvascular spasm; however, the quality of existing evidence is variable. This study aimed to systematically evaluate the efficacy and safety of anisodamine as adjuvant therapy for septic shock.

**Methods:**

We systematically searched PubMed, the Cochrane Central Register of Controlled Trials, Embase, Web of Science, CNKI, Wanfang Data, and the VIP database from inception to 1 June 2026. RCTs comparing anisodamine with control in septic shock were included. The risk of bias was assessed using the Cochrane risk-of-bias tool, and the certainty of evidence was rated using the GRADE approach.

**Results:**

A total of 14 RCTs (2 in English and 12 in Chinese) were included. Compared with control, anisodamine reduced 28-day mortality (6 studies; RR = 0.76; 95% CI, 0.62–0.93). Anisodamine also significantly increased mean arterial pressure (MD = 3.92; 95% CI, 1.85–5.98) and the systemic vascular resistance index (MD = 74.78; 95% CI, 17.59–131.97). Furthermore, anisodamine increased the Lactate clearance rate (MD = 16.27; 95% CI, 9.26–23.28), reduced serum Lactate (MD = −0.68; 95% CI, −1.10 to −0.26), and increased oxygen consumption (VO_2_; MD = 23.10; 95% CI, 11.53–34.68) without increasing oxygen delivery (DO_2_). Anisodamine also reduced the SOFA score (MD = −1.17; 95% CI, −1.95 to −0.39) and the APACHE-II score (MD = −1.50; 95% CI, −2.34 to −0.65), and shortened the duration of vasoactive agent use, ICU length of stay (LOS), and duration of mechanical ventilation, although the total hospital LOS was unchanged. Heart rate was similar between groups based on limited data. However, adverse events were under-reported across studies, and cardiac safety of anisodamine could not be reliably assessed from the available evidence.

**Conclusion:**

Anisodamine as an adjuvant therapy for septic shock may reduce 28-day mortality and improve certain macrocirculatory hemodynamic parameters, tissue perfusion, oxygen metabolism, and organ function indicators. A microcirculatory mechanism is plausible but remains unproven, as no direct microcirculatory endpoint could be pooled. In addition, given that the included studies were mostly small-sample trials with substantial heterogeneity and limited certainty of evidence, large-sample, multicenter RCTs are needed (ideally focusing more on microcirculatory indicators) to further clarify the mechanism, optimal dose, timing, and appropriate treatment population.

## 1 Introduction

Sepsis is a life-threatening organ dysfunction caused by a dysregulated host response to infection and represents a major public health challenge in the field of critical care medicine. Despite the substantial healthcare resources invested worldwide each year, the mortality of septic shock has not declined significantly over the past several decades (Luhr et al., 2019; Rudd et al., 2020), indicating that current treatment strategies still have certain limitations and that there is an urgent need to explore novel adjuvant therapies to improve patient outcomes.

At present, the latest 2026 edition of the Surviving Sepsis Campaign (SSC) guidelines explicitly recommends that the core management measures include early recognition, initiation of antimicrobial therapy within 1-h, aggressive fluid resuscitation, vasoactive agents to maintain hemodynamic stability, source control of infection, and organ function support (PRESCOTT et al., 2026). However, clinical practice has shown that, even with the support of high-dose vasoactive agents and normalization of macrohemodynamic parameters, a considerable proportion of patients do not achieve a concomitant improvement in tissue perfusion and oxygen metabolism. This suggests that the circulatory failure of septic shock is not merely an abnormality of large-vessel tone, but rather involves multilevel derangements of microvascular function, the endothelial barrier, and cellular metabolism, and that treatment strategies focusing solely on macrocirculation currently have marked limitations.

Anisodamine is an M-cholinergic receptor antagonist extracted from the solanaceous plant Anisodus tanguticus and first synthesized in China in 1975; it has been used in China for the treatment of septic shock for several decades. Microcirculatory failure in septic shock results from heterogeneous capillary perfusion and functional shunting, endothelial glycocalyx degradation, leukocyte–endothelial adhesion, platelet–leukocyte aggregation and microthrombosis, and impaired cellular oxygen extraction. Anisodamine may counteract several of these processes: as a muscarinic receptor antagonist it relieves microvascular spasm and redistributes flow toward under-perfused capillaries (Zhang Y. et al., 2023; Du et al., 2021; Zeng et al., 2021); by re-routing acetylcholine toward the α7nAChR it activates the cholinergic anti-inflammatory pathway and dampens the inflammatory response driving endothelial and glycocalyx injury (Liu et al., 2009; Zhao et al., 2011); and experimental evidence indicates it can protect the glycocalyx, inhibit platelet and granulocyte aggregation, and preserve mitochondrial function (Zhang Y. et al., 2023; Du et al., 2021; Zeng et al., 2021). These complementary effects provide the biological rationale for evaluating anisodamine as an adjuvant therapy targeting the microcirculatory component of septic shock.

However, although anisodamine as adjuvant therapy for septic shock has shown promising clinical application prospects, the existing evidence, apart from two relatively large multicenter clinical studies, is derived almost entirely from single-center, small-sample randomized controlled trials of uneven overall quality with evident limitations. On this basis, the present study employed the methods of systematic review and meta-analysis to comprehensively search and include randomized controlled trials of anisodamine as adjuvant therapy for septic shock published domestically and internationally, in order to comprehensively evaluate its clinical value and provide further evidence-based support for the clinical treatment strategy of septic shock.

## 2 Materials and methods

### 2.1 Protocol and registration

This systematic review and meta-analysis was conducted in accordance with the Preferred Reporting Items for Systematic Reviews and Meta-Analyses (PRISMA) guidelines and was registered in the PROSPERO international prospective register of systematic reviews (https://www.crd.york.ac.uk/prospero; registration number: CRD420261439261).

### 2.2 Search methods and inclusion criteria

We searched PubMed, the Cochrane Central Register of Controlled Trials, Embase, Web of Science, China National Knowledge Infrastructure (CNKI), Wanfang Data, and the VIP database. A combination of the MeSH terms used in PubMed — “Anisodamine,” “Sepsis,” and “Shock, Septic” — together with related Chinese and English free-text terms was applied (detailed search strategies are provided in Supplementary Material S1). The search covered eligible studies published from the inception of each database to 1 June 2026. After removing duplicates, two authors independently screened the titles and abstracts of the retrieved records and determined the finally included studies through full-text review. Included studies were required to meet the following criteria.

1. Participants: aged ≥15 years [1 multicenter RCT (Yu 2021), 4 single-center RCTs did not specify a lower age limit, and all remaining studies used ≥18 years. Given that the mean age of these studies was 47–72 years, the actual proportion of patients aged <15 years was extremely low, and given the relatively high quality of the Yu 2021 multicenter study, we decided to retain this study and set the lower age limit for inclusion at 15 years; the pooled result after excluding this study in the sensitivity analysis showed no appreciable change. The protocol has been registered and updated in the PROSPERO systematic review database with the reasons for modifications noted].
2. Comparison: anisodamine group versus control group
3. Diagnosis: septic shock
4. Outcome data: data for the outcome measures were obtainable
5. Study design: RCT

The inclusion criteria were determined by two authors after discussion; disagreements were first resolved through negotiation, and if consensus could not be reached, a third member made the final decision. All studies underwent comprehensive assessment before inclusion.

### 2.3 Data extraction

Two authors independently extracted the data using a data extraction form and summarized and discussed the necessity of the data. When necessary, we contacted the authors to obtain the required information. The following data were extracted from each study: first author, year of publication, type of study design, baseline characteristics of the participants, sample size, usage and dosage of anisodamine and outcome measures.

### 2.4 Quality assessment

In this study, only RCTs were included, and two authors independently assessed the risk of bias of each study using the Cochrane Collaboration risk-of-bias assessment tool. When disagreements arose, they were first resolved through negotiation, and if consensus could not be reached, a third member made the final decision.

### 2.5 Publication bias

Publication bias was planned to be assessed using funnel plots when at least ten studies contributed to an outcome. Because fewer than ten studies were available for every outcome, funnel plots are presented for reference only and were not interpreted as evidence for or against publication bias.

### 2.6 Outcome measures

The primary endpoint was 28-day mortality. The secondary endpoints included mean arterial pressure (MAP), cardiac index, systemic vascular resistance index (SVRI), lactate (Lac), Lac clearance rate, and duration of vasoactive agent use, among others.

### 2.7 Subgroup and sensitivity analyses

In this study, subgroup analyses of 28-day mortality were performed by diagnostic criteria, sample size, and risk of bias; subgroup analyses of MAP were performed by diagnostic criteria and dose; a subgroup analysis by diagnostic criteria and a sensitivity analysis were performed for Lac; subgroup analyses of safety were performed by dose and baseline heart rate (HR); and sensitivity analyses were performed for Lac clearance rate and SOFA score.

### 2.8 GRADE assessment

This study used the GRADE approach to rate the certainty of evidence for all outcomes as high, moderate, low, or very low. This approach systematically evaluates risk of bias, indirectness, inconsistency, imprecision, and publication bias. Two authors independently performed the GRADE rating; disagreements were first resolved through negotiation, and if consensus could not be reached, a third member made the final decision.

### 2.9 Statistical methods

Data analysis was performed using Review Manager (RevMan 5.4.1) as well as Microsoft Word and Excel. Results are presented as forest plots and tables, respectively. An *I* ^*2*^ > 50% or *P* < 0.05 was considered to indicate significant heterogeneity; for outcomes with high heterogeneity, subgroup or sensitivity analyses were further performed where appropriate to explore the sources of heterogeneity, and when the sources could not be analyzed or the number of studies was small, a random-effects model was used for analysis. We used the risk ratio (RR) and 95% confidence interval (CI) to compare 28-day mortality; for the remaining continuous variables, the mean difference (MD) was used as the summary statistic when the method of measurement and units were the same, and the standardized mean difference (SMD) was used when the outcome variables were inconsistent across studies or the measurement units differed. Outcomes with extreme heterogeneity (*I* ^*2*^ > 90%) that could not be explained by subgroup or sensitivity analyses are reported narratively, with any pooled value presented for reference only.

## 3 Results

### 3.1 Literature search results and risk-of-bias assessment

The initial search identified 1,910 records. After deduplication and screening of titles and abstracts, 36 records were selected, and after excluding retrospective studies, studies that did not include the primary or secondary endpoints, and studies for which data could not be obtained, 14 studies were finally included. The study selection process and the risk-of-bias assessment are shown in Figure 1 and Supplementary Material S2.

**FIGURE 1:**
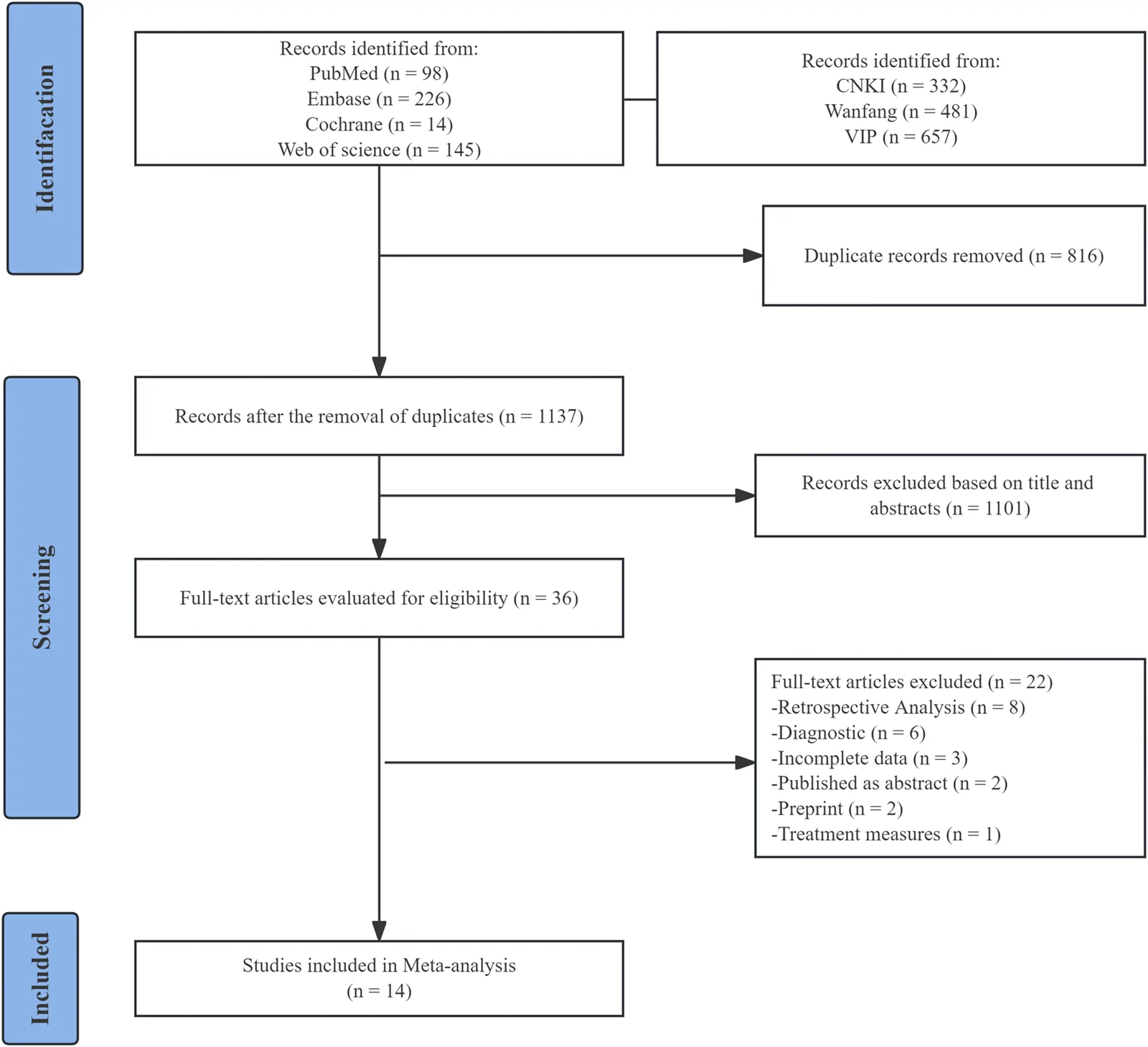
Study flow diagram.

### 3.2 Publication bias

The funnel plots for 28-day mortality, MAP, Lac, and HR in the anisodamine and control groups are shown in Supplementary Material S3 for reference.

### 3.3 Basic characteristics of the included studies

A total of 2 English-language (Zhang F. et al., 2023; Yu et al., 2021) and 12 Chinese-language articles were included (Wang and Liu, 2025; Jia et al., 2026; Peng et al., 2024; Gong et al., 2008; Qin et al., 2020; Xu et al., 2012; Liu et al., 2008; Zou et al., 2013; Fang et al., 2017; Wu et al., 2021; LJ, 2021; Hou et al., 2024), all of which were RCTs. The basic characteristics of the included studies are shown in Table 1.

**TABLE 1:** Characteristics of included randomized controlled trials.

Study	Year	Design	Diagnostic criteria	Anisodamine group/Control group				Anisodamine dosage	Outcomes
				Sample size (Treatment/Control)	Male/%	Age/yr	Treatment measures		
Gong P	2008	RCT	Septic shock 1991 Sepsis-1	15/15	Overall 55.6	Overall 49.0 ± 18.7	Anisodamine + conventional therapy/conventional therapy	Initial 10–40 mg IV, additional 1/2–1 dose q15-30min prn; after achieving “anisodaminization” within 1 h, maintained at half-dose q0.5 h	MAP, HR
Liu YS	2008	RCT	Septic shock 1991 Sepsis-1	21/21	71.4/57.1	50.21 ± 4.23 /48.01 ± 3.54	Anisodamine + conventional therapy/conventional therapy	1.2–4.0 mg/(kg·d) continuous IV infusion; tapered and discontinued after hemodynamic stabilization and vasopressor withdrawal	28-day mortality Lac
Xu J	2012	RCT	Septic shock 1991 Sepsis-1	24/22	Overall 70.2	Overall 59.17 ± 14.17	Anisodamine + conventional therapy/conventional therapy	Continuous IV infusion of anisodamine 2 mg/h	MAP, HR
Zou YC	2013	RCT	Septic shock 1991 Sepsis-1	32/31	62.5/67.7	53.1 ± 18.2 /50.7 ± 17.6	Anisodamine + conventional therapy/conventional therapy	After fluid resuscitation within 6 h, anisodamine micro-pump infusion (0.1–0.5) mg/(kg·h)	28-day mortality Lac, Lac clearance, SOFA, ICU LOS, vasopressor duration, MV duration
Fang G	2017	RCT	Septic shock 1991 Sepsis-1	66/66	54.5/53.0	55 ± 10/53 ± 12	Anisodamine + conventional therapy + dopamine/conventional therapy + dopamine	Continuous IV infusion at 10 μg/(kg·min)	CI, SVRI, DO_2_, VO_2_, HR
Qin G	2020	RCT	Septic shock 2016 Sepsis-3	30/32	80.0/65.6	72.27 ± 12.25 /69.13 ± 15.30	Anisodamine + conventional therapy/conventional therapy	10 mg IV q15min, within the limit of no “atropinization”, for 48 h	28-day mortality MAP, Lac, CI, SVRI
Ren LJ	2021	RCT	Septic shock 2001 Sepsis-2	35/36	62.9/63.9	54.87 ± 7.15 /54.62 ± 7.06	Anisodamine + conventional therapy + dopamine/conventional therapy + dopamine	40 mg in 5% glucose 250 mL IV drip, repeated q30min for 12 h	CI, SVRI, DO_2_, VO_2_, HR
Wu SQ	2021	RCT	Septic shock 2016 Sepsis-3	80/80	55.0/53.8	46 ± 7/45 ± 8	Anisodamine + conventional therapy/conventional therapy	Started at 0.1 mg/(kg·h), gradually increased to max 0.5 mg/(kg·h); maintained at original dose for 6 h after rewarming and CRT <3 s, then tapered	MAP, Lac, Lac clearance
Yu	2021	RCT	Septic shock 2001 Sepsis-2	181/174	62/60	69 (58.75, 79) /67 (56, 78)	Anisodamine + conventional therapy/conventional therapy	Initial 10 mg IV, maintenance 0.1–0.5 mg/(kg·h) continuous IV infusion	28-day mortality Lac, hospital LOS, SOFA, ICU LOS, vasopressor duration, MV duration
Zhang	2023	RCT	Septic shock 2016 Sepsis-3	203/201	63.05/63.18	59.19 ± 10.56 /58.31 ± 12.03	Anisodamine + conventional therapy/conventional therapy	Initial 0.5 mg/kg IV (min 20 mg, max 40 mg) over 3 min; maintenance 0.02–0.1 mg/(kg·h) continuous IV infusion, max 200 mg/d; discontinued after shock reversal	28-day mortality Lac, Lac clearance, SOFA, hospital LOS, vasopressor duration
Hou CC	2024	RCT	Septic shock 2016 Sepsis-3	51/51	58.8/64.7	52 ± 9/52 ± 8	Anisodamine + conventional therapy + dopamine/conventional therapy + dopamine	40 mg IV q30min, adjusted at physician’s discretion, for 12 h	HR, DO_2_, VO_2_
Peng Y	2024	RCT	Septic shock 2016 Sepsis-3	36/36	69.44/47.22	70.17 ± 11.99 /66.53 ± 13.64	Anisodamine + conventional therapy/conventional therapy	Initial 10 mg IV, maintenance 0.1–0.5 mg/kg/h continuous IV infusion for 7 d	28-day mortality APACHE-II, hospital LOS, ICU LOS
Wang XM	2025	RCT	Septic shock 2016 Sepsis-3	55/55	58.2/54.5	60.19 ± 5.25 /60.23 ± 5.11	Anisodamine + conventional therapy/conventional therapy	Initial 10 mg IV q15min; dose escalated by 10 mg per administration if fluid resuscitation target not reached; max single dose ≤40 mg	MAP, SOFA, APACHE-II, ICU LOS, vasopressor duration, MV duration
Jia MY	2026	RCT	Septic shock 2016 Sepsis-3	131/131	57.3/60.3	57.97 ± 10.01 /59.29 ± 7.33	Anisodamine + conventional therapy + Norepinephrine/conventional therapy + Norepinephrine	10 mg IV q10min until shock reversal (expected 24–48 h), followed by 24 h observation	HR

APACHE-II, Acute Physiology and Chronic Health Evaluation II; CI, cardiac index; CRT, capillary refill time; DO_2_, oxygen delivery; HR, heart rate; ICU, intensive care unit; IV, intravenous; Lac, serum lactate; LOS, length of stay; MAP, mean arterial pressure; MV, mechanical ventilation; RCT, randomized controlled trial; Sepsis-1 (1991 ACCP/SCCM), Sepsis-2 (2001 SCCM/ESICM/ACCP/ATS/SIS), Sepsis-3 (2016 SCCM/ESICM); SOFA, sequential organ failure assessment; SVRI, systemic vascular resistance index; VO_2_, oxygen consumption.

### 3.4 Primary endpoint

Among the included studies, 6 reported 28-day mortality. Compared with the control group, the anisodamine group significantly reduced 28-day mortality in patients with septic shock (RR = 0.76; 95% CI, 0.62–0.93; P for heterogeneity = 0.34; I^2^ = 12%). Subgroup analysis by diagnostic criteria showed Sepsis-1/2 (RR = 0.78; 95% CI, 0.56–1.10; P for heterogeneity = 0.27; I^2^ = 24%) and Sepsis-3 (RR = 0.69; 95% CI, 0.52–0.90; P for heterogeneity = 0.49; I^2^ = 0%), with no statistically significant difference between subgroups (P = 0.54) (Figure 2A). Subgroup analysis by risk-of-bias grade showed low risk of bias (RR = 0.83; 95% CI, 0.67–1.04; P for heterogeneity = 0.23; I^2^ = 30%) and moderate/high risk of bias (RR = 0.53; 95% CI, 0.35–0.82; P for heterogeneity = 0.91; I^2^ = 0%); the test for subgroup differences did not reach statistical significance (P = 0.07), and, although the moderate/high risk-of-bias subgroup showed a numerically larger effect, this difference should be interpreted with caution given the non-significant test for subgroup differences and the small number of studies (Figure 2B). (The subgroup analysis of multicenter versus single-center studies yielded results consistent with the risk-of-bias grading).

**FIGURE 2:**
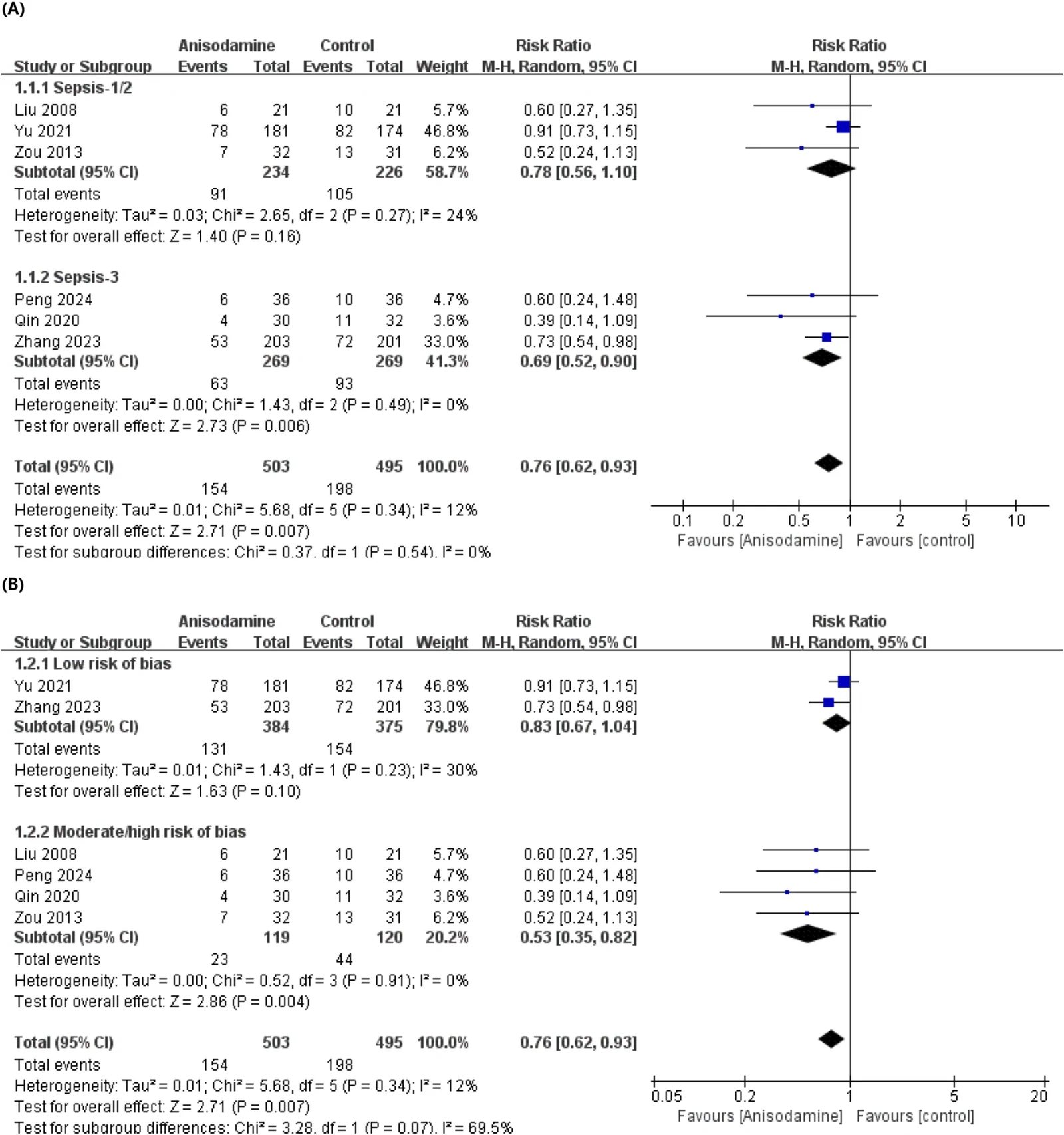
28-day mortality: **(A)** Forest plot of 28-day mortality in anisodamine vs. control, stratified by diagnostic criteria; **(B)** Forest plot of 28-day mortality in anisodamine vs. control, stratified by risk of bias.

### 3.5 Secondary endpoints

#### 3.5.1 Macrohemodynamics

In this study, MAP, cardiac index, and SVRI were collectively regarded as macrohemodynamic parameters. Compared with the control group, the anisodamine group significantly increased MAP in patients with septic shock (MD = 3.92; 95% CI, 1.85–5.98; P for heterogeneity = 0.21, I^2^ = 32%). Subgroup analysis of MAP by diagnostic criteria showed Sepsis-1/2 (MD = 7.22; 95% CI, 3.00–11.45; P for heterogeneity = 0.96; I^2^ = 0%) and Sepsis-3 (MD = 2.87; 95% CI, 2.08–3.66; P for heterogeneity = 0.49; I^2^ = 0%), with no statistically significant difference between subgroups (P = 0.23) (Figure 3A). Subgroup analysis by dose showed high dose >0.1 mg/(kg·h) (MD = 4.43; 95% CI, 1.17–7.69; P for heterogeneity = 0.43, I^2^ = 0%) and low dose <0.1 mg/(kg·h) (MD = 4.43; 95% CI, 0.21–8.64; P for heterogeneity = 0.06, I^2^ = 32%), with no statistically significant difference between groups (P = 1.00) (Supplementary Material S4). SVRI (MD = 74.78; 95% CI, 17.59–131.97; P for heterogeneity = 0.12, I^2^ = 53%) is shown in Figure 3B. Cardiac index (MD = −0.02; 95% CI, −0.64–0.61; P for heterogeneity <0.00001, I^2^ = 95%) exhibited extreme and unexplainable heterogeneity, rendering the pooled estimate uninterpretable. This outcome is therefore summarized narratively, and the reference-only pooled estimate suggested no evident difference between the two groups (Figure 3C).

**FIGURE 3:**
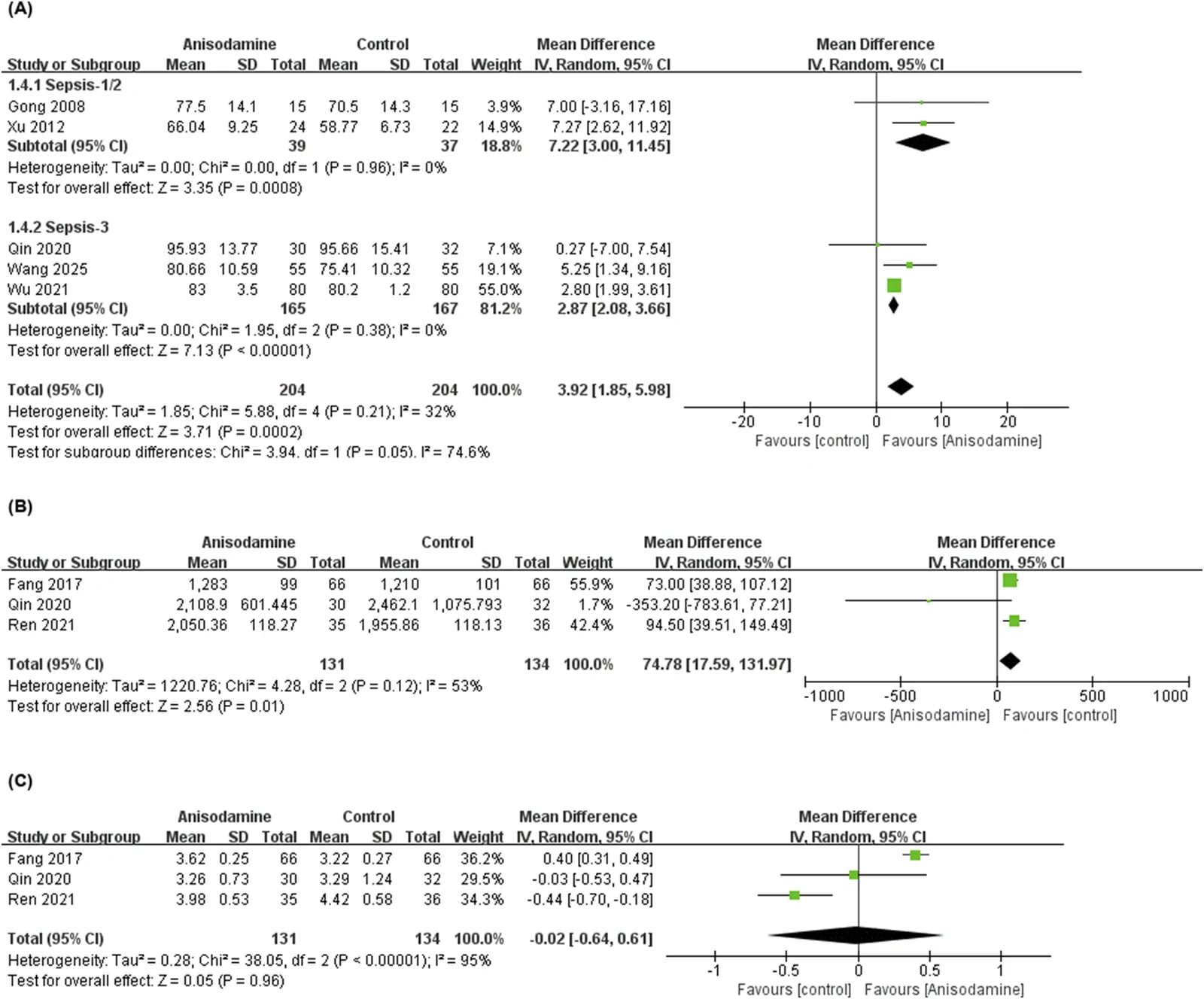
Macrohemodynamic Parameters: **(A)** Forest plot of MAP in anisodamine vs. control, stratified by diagnostic criteria; **(B)** Forest plot of Systemic Vascular Resistance Index; **(C)** Forest plot of Cardiac Index.

#### 3.5.2 Tissue perfusion

In this study, Lac and Lac clearance rate were used as parameters to measure tissue perfusion. Compared with the control group, the anisodamine group significantly increased the Lac clearance rate (MD = 16.27; 95% CI, 9.26–23.28; P for heterogeneity = 0.0004, I^2^ = 87%) (Figure 4A) and significantly reduced the Lac level in patients with septic shock (MD = −0.68; 95% CI, −1.10 to −0.26; P for heterogeneity = 0.001, I^2^ = 75%). Subgroup analysis of Lac by diagnostic criteria showed Sepsis-1/2 (MD = −0.63; 95% CI, −1.56–0.29; P for heterogeneity = 0.009; I^2^ = 79%) and Sepsis-3 (MD = −0.77; 95% CI, −1.18 to −0.36; P for heterogeneity = 0.06; I^2^ = 64%), with no statistically significant difference between subgroups (P = 0.80) (Figure 4B).

**FIGURE 4:**
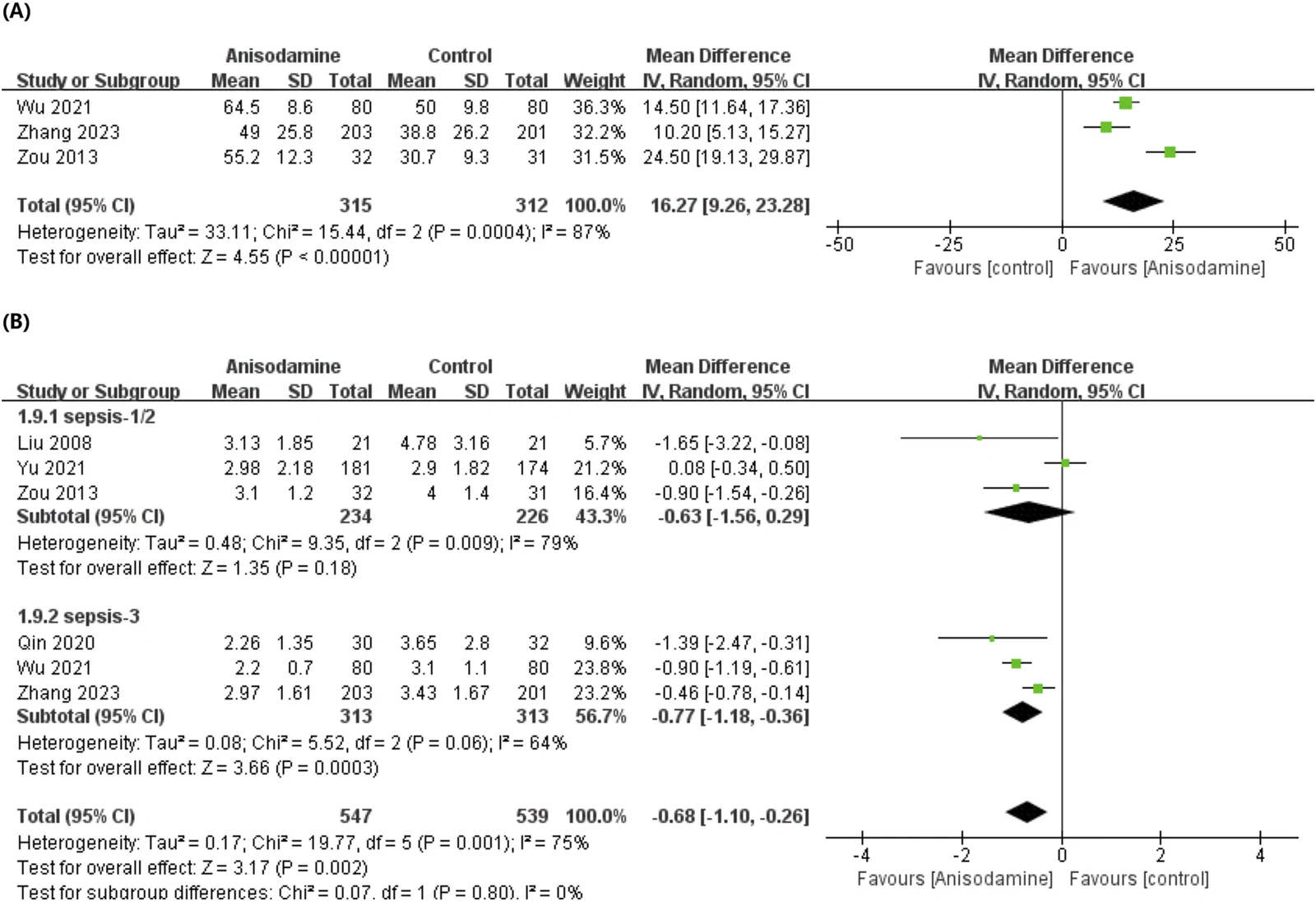
Tissue Perfusion Parameters: **(A)** Forest plot of Lac clearance Rate; **(B)** Forest plot of Lac in anisodamine vs. control, stratified by diagnostic criteria.

#### 3.5.3 Oxygen metabolism

This study included two indicators, oxygen delivery (DO_2_) and oxygen consumption (VO_2_), to evaluate changes in tissue oxygen metabolism. The results showed that the anisodamine group had no significant impact on DO_2_ (MD = 10.96; 95% CI, −60.18–82.10; P for heterogeneity <0.00001, I^2^ = 97%), this pooled estimate is reported for reference only; given this extreme heterogeneity, the pooled estimate of DO_2_ is reported for reference only, but significantly increased tissue VO_2_ (MD = 23.10; 95% CI, 11.53–34.68; P for heterogeneity = 0.03, I^2^ = 72%) (Figure 5).

**FIGURE 5:**
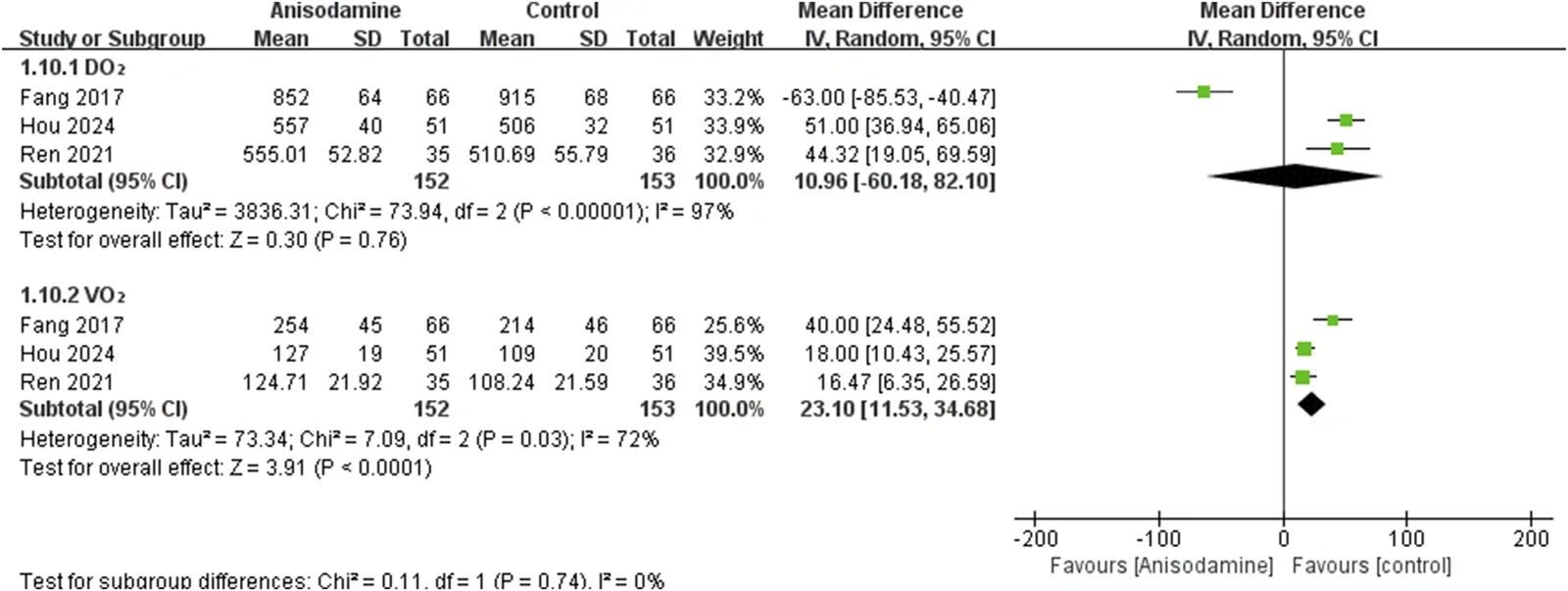
Forest plot for Oxygen Delivery and Oxygen Consumption.

#### 3.5.4 Organ function and disease severity scores

In this study, the SOFA score and the APACHE-II score were used as indicators of patient organ function and disease severity, respectively. The results showed that the anisodamine group significantly reduced the SOFA score (MD = −1.17; 95% CI, −1.95 to −0.39; P for heterogeneity = 0.04, I^2^ = 65%) (Figure 6) and the APACHE-II score (MD = −1.50; 95% CI, −2.34 to −0.65; P for heterogeneity = 0.42, I^2^ = 0%) (Figure 6).

**FIGURE 6:**
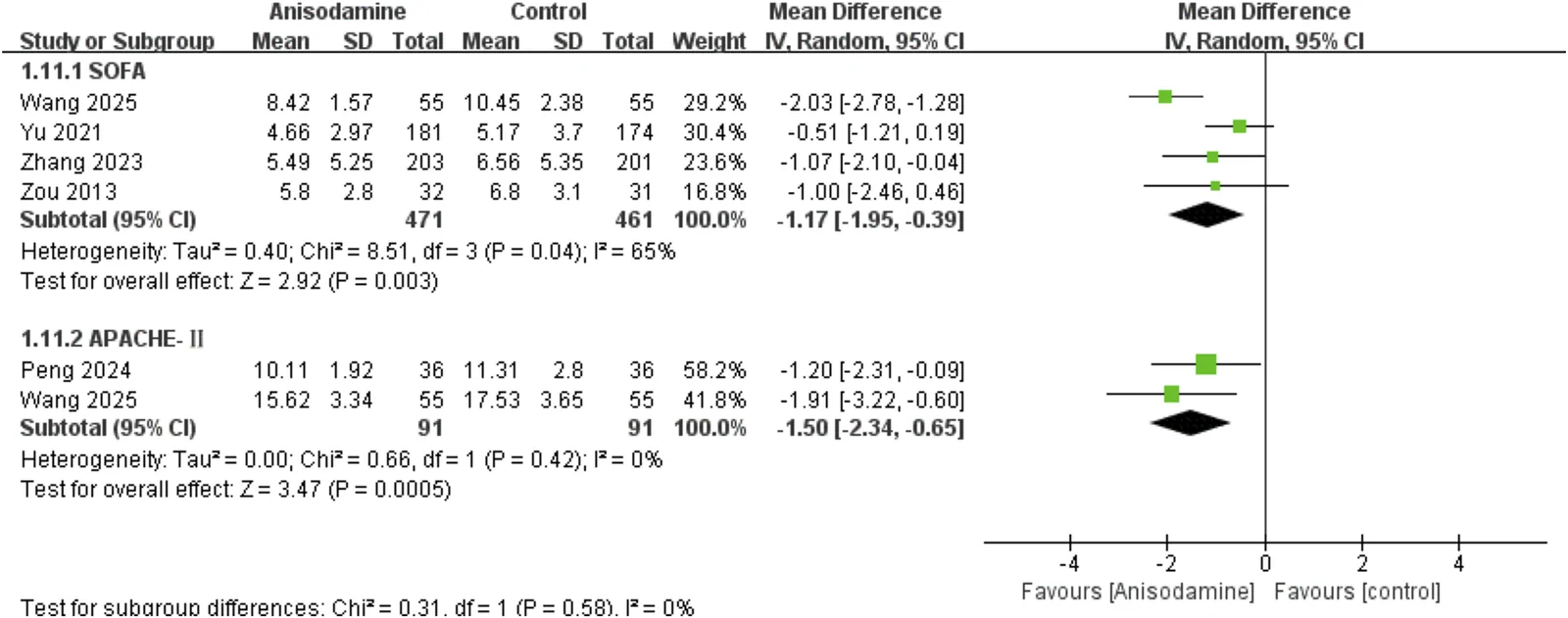
Forest plot for SOFA Score and APACHE-II Score.

#### 3.5.5 Clinical treatment-related outcomes

In this study, total hospital length of stay (LOS), ICU LOS, duration of vasoactive agent use, and duration of mechanical ventilation (MV) were collectively used as indicators of the clinical recovery process. The results showed that anisodamine did not reduce the total hospital LOS (MD = −2.06; 95% CI, −5.63–1.50; P for heterogeneity <0.00001, I^2^ = 92%), this pooled estimate is reported for reference only; however, anisodamine significantly reduced the duration of vasoactive agent use (MD = −0.89; 95% CI, −1.03 to −0.76; P for heterogeneity = 0.97, I^2^ = 0%), the ICU LOS (MD = −1.57; 95% CI, −2.85 to −0.29; P for heterogeneity = 0.03, I^2^ = 67%), and the duration of MV (MD = −1.19; 95% CI, −1.69 to −0.70; P for heterogeneity = 0.90, I^2^ = 0%) (Figure 7).

**FIGURE 7:**
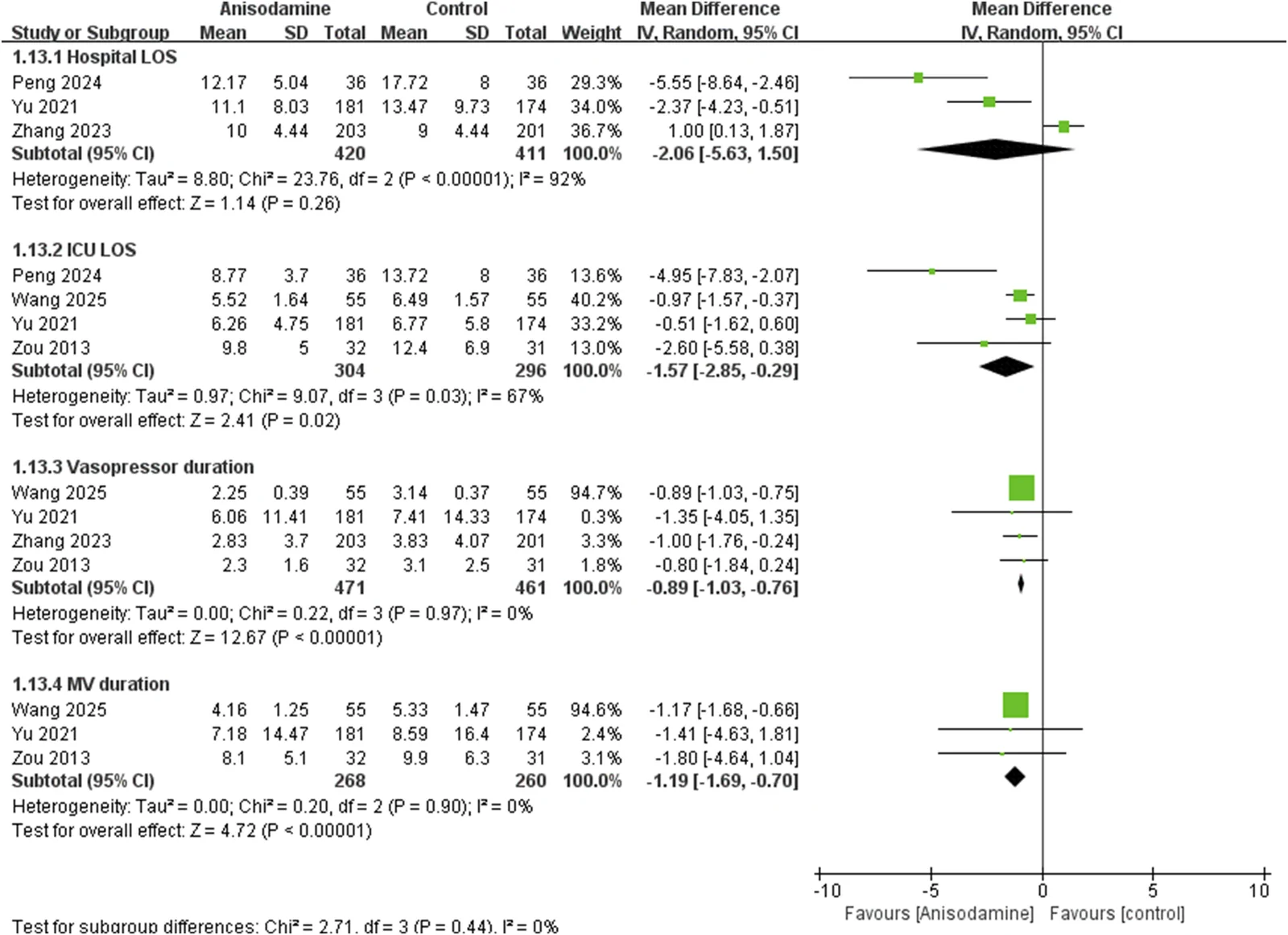
Forest plot for Clinical Recovery Outcomes (Hospital LOS, ICU LOS, Vasopressor duration, and MV duration).

#### 3.5.6 Safety

Owing to the limited data of the included studies, only HR was included as a safety outcome in this study. HR was analyzed as a continuous safety-related physiological parameter rather than as a discrete adverse event. The results showed that the anisodamine group did not significantly increase HR (MD = −5.18; 95% CI, −13.53–3.17; P for heterogeneity <0.00001, I^2^ = 98%), this pooled estimate is reported for reference only. And the dose and baseline-HR subgroup analyses below (also I^2^ = 92–98%) should be read as exploratory rather than confirmatory. Subgroup analysis showed high dose >0.1 mg/(kg·h) (MD = −7.32; 95% CI, −15.98–1.34; P for heterogeneity <0.00001, I^2^ = 98%) and low dose <0.1 mg/(kg·h) (MD = 6.01; 95% CI, −0.44–12.46; heterogeneity not applicable), with a significant test for subgroup differences (P = 0.02) (Supplementary Material S5), as well as high baseline HR > 125 bpm (MD = −14.09; 95% CI, −19.57 to −8.61; P for heterogeneity <0.00001, I^2^ = 94%) and low baseline HR < 125 bpm (MD = 14.03; 95% CI, −1.64–29.70; P for heterogeneity = 0.0005, I^2^ = 92%), with a significant difference between groups (P = 0.0009) (Figure 8). As for overall adverse events such as arrhythmia, urinary retention, and blurred vision, no significant difference was observed between the anisodamine group and the control group; however, due to the limited data, only a narrative analysis was performed (see Supplementary Material S6).

**FIGURE 8:**
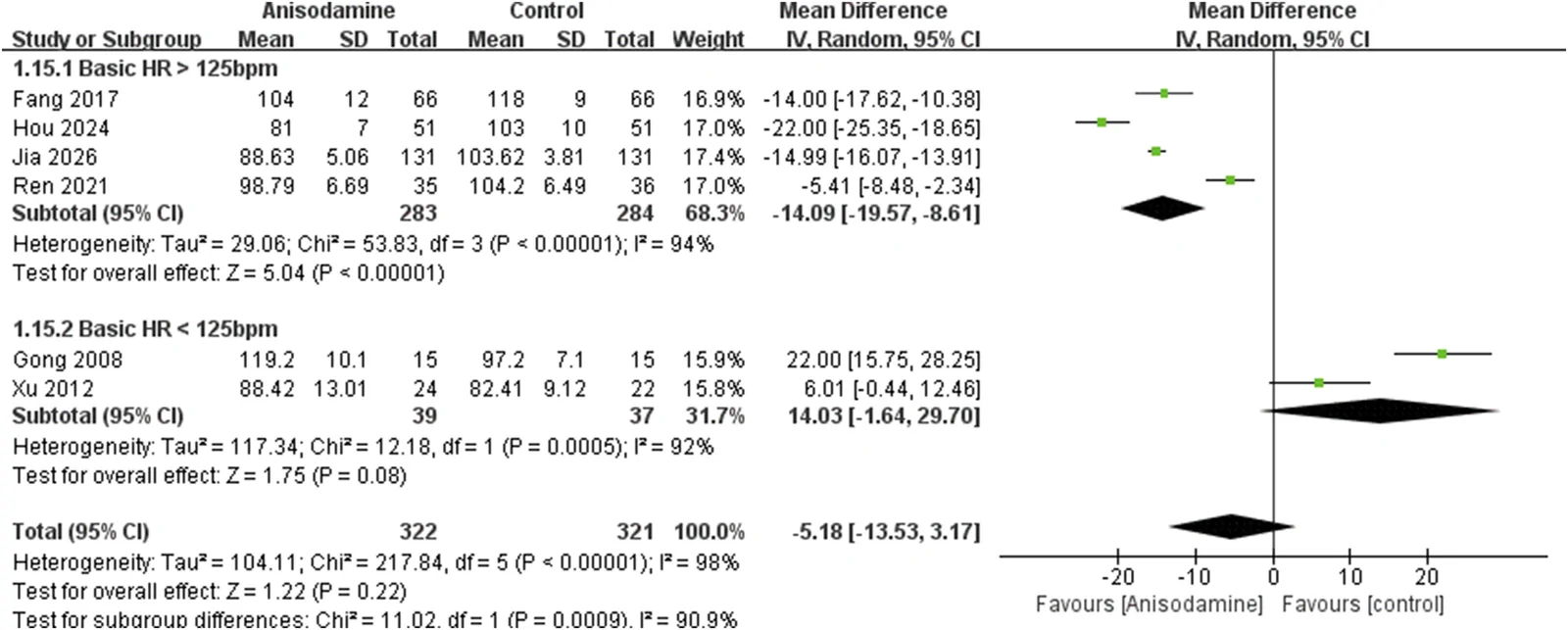
Forest plot of heart rate in anisodamine vs. control, stratified by baseline HR.

### 3.6 Sensitivity analyses

In the sensitivity analysis of Lac, after excluding the study by Yu 2021, the Sepsis-1/2 subgroup showed a significant reduction in Lac (MD = −1.01; 95% CI, −1.60 to −0.41; P for heterogeneity = 0.39; I^2^ = 0%), the overall result remained robust, the heterogeneity disappeared (I^2^ = 43%), and there was no statistically significant difference between subgroups (P = 0.51) (Supplementary Material S7A). After removing Wang 2025 from the sensitivity analysis of SOFA scores, the heterogeneity disappeared (I^2^ = 0%) (Supplementary Material S7B). For all results involving the Yu 2021 study, the pooled results after excluding this study showed no appreciable change (Supplementary Material S8). Given that the results of the vasopressor and MV duration analyses were substantially influenced by Wang 2025, leave-one-out sensitivity analyses were performed. The results showed that for vasopressors, the findings remained significant after excluding each study one at a time (Supplementary Material S9); for MV duration, after excluding Wang 2025, the pooled effect size became (MD = −1.63; 95% CI, −3.76 to 0.50; P for heterogeneity = 0.86; I^2^ = 0%), while the results remained unchanged after sequentially excluding each of the remaining studies (Supplementary Material S10).

### 3.7 GRADE assessment

The certainty of evidence for the various outcomes ranged from very low to moderate. The detailed GRADE assessment table is provided in Supplementary Material S11.

## 4 Discussion

### 4.1 Main findings

This systematic review and meta-analysis aimed to analyze the effect of anisodamine as adjuvant therapy on clinical outcomes in patients with septic shock. The pooled results of the included studies showed that, compared with conventional treatment, anisodamine adjuvant therapy was associated with a reduction in 28-day mortality in patients with septic shock and improved some macrohemodynamic, tissue perfusion, and oxygen metabolism indicators. Compared with previous studies, the present study focused not only on traditional circulatory indicators but also included indicators related to tissue perfusion, oxygen metabolism, organ function, and clinical recovery; this comprehensive analysis may help to elucidate the potential mechanisms of action of anisodamine in septic shock.

Regarding the pathophysiological mechanisms of septic shock, current research mainly includes uncontrolled inflammatory responses caused by infection and even immunosuppression, vascular hyporeactivity, vascular endothelial cell injury and glycocalyx degradation, microcirculatory dysfunction caused by microthrombus formation, impaired tissue oxygen utilization, mitochondrial dysfunction, and septic cardiomyopathy (Angus and Van Der Poll, 2013; Hotchkiss et al., 2016; Lelubre and Vincent, 2018). Although the core management measures recommended by the SSC can maintain macrohemodynamic stability, a considerable proportion of patients still exhibit tissue hypoxia and a dissociation between the macrocirculation and microcirculation in terms of microcirculatory and oxygen metabolism disorders; that is, even when patients reach the target MAP value, reduced capillary perfusion and tissue hypoxia may still be present. Anisodamine is a natural alkaloid with anticholinergic activity, and previous studies have shown that it can activate the cholinergic anti-inflammatory pathway and inhibit the production of pro-inflammatory cytokines (Liu et al., 2009; Zhao et al., 2011). This effect is parallel to the relief of microvascular spasm. Together, these effects improve vascular paralysis and promote the restoration of tissue oxygen utilization, ultimately improving prognosis. However, the randomized controlled trials included in this meta-analysis rarely report immune or inflammatory endpoints, so the immunomodulatory effect of anisodamine cannot be tested in the meta-analysis.

### 4.2 Organ function and clinical outcomes

The results of this systematic review and meta-analysis showed that the anisodamine group significantly reduced the SOFA score (heterogeneity disappeared after sensitivity analysis, which was considered to be caused by the markedly higher baseline SOFA score of the patients included in Wang 2025 compared with the other three studies) and the APACHE-II score, while also reducing the duration of vasoactive agent use and the ICU LOS.

The SOFA score mainly reflects the degree of dysfunction of multiple organs in patients with sepsis. A meta-analysis by Jain et al. helps to further clarify our results, showing that anisodamine can reduce the incidence of contrast-induced nephropathy and improve serum creatinine and eGFR at 24 h and 48 h (Jain et al., 2024). The multicenter study by Zhang et al., included in the present analysis, showed that in patients with septic shock the anisodamine group improved blood urea nitrogen and serum creatinine at 48 h and 72 h, and that at 72 h and 7 days the CRRT weaning rate in the anisodamine group was significantly higher than in the control group (Zhang F. et al., 2023); this is primarily attributable to its effects on improving renal perfusion and related mechanisms. A study by Peng et al. found that anisodamine treatment can improve PLT and D-dimer levels in patients with septic shock (Peng et al., 2024). These results all support, to some extent, the finding of an improved SOFA score in the present meta-analysis. The APACHE-II score is an important indicator for the comprehensive evaluation of disease severity and mortality risk, and its decrease further reflects that anisodamine may improve the overall disease state of patients and reduce their risk of death. Although our pooled analysis also suggested a shorter duration of mechanical ventilation in the anisodamine group, the leave-one-out sensitivity analysis indicated that this finding was not robust. Specifically, for the duration of mechanical ventilation, after excluding Wang 2025, the pooled effect size became (MD = −1.63; 95% CI, −3.76–0.50; P for heterogeneity = 0.86; I^2^ = 0%), whereas the results remained unchanged after sequentially excluding each of the remaining studies. Given that only 3 studies were included, the leave-one-out analysis pooled only 2 studies at a time, and the estimates were inherently unstable. Therefore, these results should be interpreted with caution and warrant further verification in additional high-quality studies.

### 4.3 Possible mechanisms of anisodamine

Notably, this study found that adjuvant anisodamine therapy significantly increased MAP and SVRI in patients with septic shock, but did not significantly improve cardiac index, suggesting that its pressor strategy is not primarily mediated by enhancing cardiac function.

To explore the possible reasons, we further analyzed Lac, Lac clearance rate, DO_2_, and VO_2_ within the limits of the available data. This study found that anisodamine treatment could significantly reduce the blood Lac level and increase the Lac clearance rate. Lac is an important indicator for evaluating the tissue perfusion state and resuscitation effect in patients with sepsis. During the course of septic shock, even after systemic DO_2_ is restored, regional tissues may remain in a hypoxic state, leading to persistently elevated Lac levels. Therefore, a decrease in Lac level and an improvement in Lac clearance capacity usually indicate an improvement in tissue perfusion and metabolic state. However, a decrease in Lac level cannot fully prove restoration of the microcirculation, because Lac metabolism is also affected by factors such as hepatic and renal function, inflammatory status, and cellular metabolic level (Hernandez et al., 2019; Puskarich et al., 2016). Therefore, the Lac-lowering effect of anisodamine is more likely the result of multiple factors acting together. In the sensitivity analysis for serum Lac, heterogeneity was eliminated after excluding Yu 2021, which may be attributable to the lower baseline Lac levels in that study population.

In addition, we observed that anisodamine could improve the tissue oxygen utilization capacity of patients by increasing VO_2_ without increasing DO_2_. This suggests that in septic shock, even though macroscopic oxygen supply can be largely maintained to a certain extent in most patients through combination therapy, abnormal microcirculatory blood flow distribution and cellular metabolic disorders mean that tissue cells and organs still cannot effectively utilize oxygen even when the oxygen supply is adequate, resulting in this macro–micro dissociation of oxygen utilization (Ince, 2015; Ince and Mik, 1985). Excessive increases in oxygen supply sometimes fail to improve tissue hypoxia and may instead increase the risk of cardiac load and volume overload (Rowan et al., 2017). This finding is consistent with the improvement in Lac metabolism; however, because no direct microcirculatory indices could be pooled, any inference of a microcirculation-improving effect remains indirect and should be interpreted cautiously.

The shortened duration of vasoactive agent use may partly reflect improved tissue perfusion and macrohemodynamic recovery, although it is an indirect indicator and does not by itself establish a microcirculatory benefit. When tissue perfusion is restored and vascular reactivity improves, the patient’s dependence on vasopressor agents decreases, and clinicians are able to reduce and discontinue vasoactive agents earlier.

However, it cannot be ignored that, owing to the limited number of included studies, this study was unable to include microcirculatory indicators such as perfused vessel density (PVD), proportion of perfused vessels (PPV), and capillary refill time (CRT) in the meta-analysis. Nevertheless, some studies have found that PVD and PPV in the anisodamine group were significantly higher than in the control group (Wang and Liu, 2025). Two retrospective studies by Ma et al. and Hong et al. found that the anisodamine group could significantly improve the CRT at 24 h and 72 h (Hong et al., 2024; Ma et al., 2025). Furthermore, animal experiments have also demonstrated that anisodamine can improve mesenteric microcirculation (Zhong et al., 2023).

Combining the above results from the limited microcirculatory indicators, we consider that anisodamine, as a cholinergic receptor antagonist, may—under the support of vasoactive agents—restore SVRI and improve MAP by improving vascular reactivity. A parallel improvement in microvascular blood flow is plausible but could not be confirmed in this meta-analysis because microcirculatory indices were not pooled. Furthermore, the lack of a significant difference in cardiac index, along with substantial heterogeneity, may be attributable to the limited number of included studies and the considerable variability in patients' baseline cardiac function, measurement methods, and assessment time points; therefore, interpretation of the cardiac index findings warrants caution.

Although anisodamine can improve several secondary endpoints, this study did not find a positive effect of anisodamine on the total hospital LOS, possibly because the total hospital LOS in septic shock is influenced by multiple factors such as underlying disease, infection control, and medical resources, and a single adjuvant therapeutic agent is unlikely to produce a significant effect.

### 4.4 Safety

Regarding the safety of anisodamine treatment, only 4 studies provided reports (Zhang F. et al., 2023; Yu et al., 2021; Wang and Liu, 2025; Jia et al., 2026). This study analyzed HR and found that anisodamine did not significantly increase HR, but the heterogeneity was marked. Baseline HR subgroup analysis showed that in patients with a baseline HR > 125 bpm, HR decreased significantly after the use of anisodamine, whereas patients with a baseline HR < 125 bpm and those in the dose subgroup analysis showed no significant difference. Because the high-HR and high-dose subgroups of the included studies overlapped highly, this study was unable to distinguish their independent effects. Considering the pathophysiological factors, this may be because septic shock causes a pathological/compensatory increase in HR, and the HR decreases secondarily after the underlying cause is relieved by anisodamine adjuvant therapy; however, the above is an exploratory analysis with extreme heterogeneity (I^2^ = 94–98%) and still requires validation by prospective studies. Across the four studies reporting safety outcomes (including two multicenter trials), the overall incidence of adverse events—such as arrhythmia, urinary retention, and blurred vision—did not differ significantly between the anisodamine and control groups. Furthermore, the dosage, administration regimen, and timing of anisodamine discontinuation varied considerably across studies, which is closely related to safety. Given the anticholinergic profile of anisodamine, the possibility of cardiac adverse effects cannot be excluded and further research is warranted.

### 4.5 Clinical significance

The results of this study provide the following reference for the clinical positioning of anisodamine:

Patient selection: In theory, the patients with septic shock most likely to benefit from anisodamine are those who, despite adequate fluid resuscitation and vasoactive agent support, still have persistently elevated Lac and macro–micro dissociation (e.g., MAP at target but microcirculatory dysfunction or tissue hypoxia not improved). However, none of the existing studies enriched patients on the basis of microcirculatory status, and future randomized clinical trials should be designed to study microcirculatory changes after anisodamine treatment.

Dosing regimen: The dosing regimens of the included studies varied considerably, and the timing and duration of administration were inconsistent, so an optimal regimen cannot currently be recommended on the basis of the existing evidence.

Clarifying the positioning of anisodamine as an adjuvant to first-line vasoactive agents such as norepinephrine: On the premise that norepinephrine maintains macrohemodynamic stability, anisodamine may exert a synergistic effect by potentially improving tissue perfusion and microcirculatory distribution; however, a direct microcirculatory benefit has not yet been established.

## 5 Limitations

This study has the following limitations, which should be fully considered when interpreting the results. First, the number of included studies was limited, and most were single-center studies from a single country, which limits the generalizability of the results. Moreover, all included trials were conducted in China and the majority were published in Chinese; therefore, language and regional publication bias cannot be excluded; Second, although a random-effects model was used, the dose, timing, duration, and combination regimens of anisodamine varied considerably among studies, and the measurement time points of each indicator also differed, resulting in a certain degree of heterogeneity. Third, most of the included studies had small sample sizes and inadequate reporting of randomization and blinding, entailing a certain risk of selection bias. Fourth, some continuous-variable indicators were inadequately reported; for example, microcirculation-related parameters were reported in only a few studies and therefore could not be meta-analyzed, further studies are required to verify these findings and explore the underlying mechanisms. Fifth, inflammatory and immune endpoints were reported in very few of the included trials, precluding a meta-analysis of the immunomodulatory effects of anisodamine; future studies should incorporate such endpoints to verify the proposed cholinergic anti-inflammatory mechanism.

## 6 Conclusion

The factors determining clinical outcomes in patients with septic shock are complex and multifaceted. This meta-analysis found that anisodamine as adjuvant therapy was associated with reduced 28-day mortality and with improvements in several macrohemodynamic, tissue-perfusion (lactate), and oxygen-metabolism parameters. Whether these benefits are mediated by an improvement in the microcirculation remains unproven, because no direct microcirculatory index could be quantitatively pooled. And the included studies were predominantly small-sample trials with considerable heterogeneity in dosage, administration regimen, timing, and disease severity. Therefore, future large-sample, multicenter, rigorously designed randomized controlled trials are needed. These trials should aim to determine the optimal timing, dosing strategy, and target population for anisodamine, investigate its mechanisms using a range of microcirculatory parameters, as well as inflammatory and immune endpoints to assess its immunomodulatory mechanism, and ultimately establish its therapeutic role in septic shock.

## Data Availability

The raw data supporting the conclusions of this article will be made available by the authors, without undue reservation.
