# Supplementary material for: Efficacy and safety of anisodamine as adjuvant therapy in septic shock: a systematic review and meta-analysis
Source: Front Pharmacol. 2026 Oct 30;17:1976742. doi: 10.3389/fphar.2026.1976742 (PMC13760278; doi:10.3389/fphar.2026.1976742)
Supplement: Supplementary file 1 [file DataSheet1.pdf]

S1 Full Search Strategies for All Databases

Search date: from inception to June 1,2026. No language restrictions applied.

1. PubMed

Search	Query
**#1**	"anisodamine" [Mesh] OR anisodamine [tiab] OR "anisodamine hydrobromide" [tiab] OR racanisodamine [tiab] OR "6-hydroxyhyoscyamine" [tiab] OR "654-2" [tiab] OR "6542" [tiab] OR "654 2" [tiab] OR "654-1" [tiab] OR "6541" [tiab] OR "654 1" [tiab]
**#2**	"Sepsis" [Mesh] OR sepsis [tiab] OR "bloodstream infection" [tiab] OR "bloodstream infections" [tiab] OR septicemia [tiab] OR septicemias [tiab] OR "blood poisoning" [tiab] OR "blood poisonings" [tiab] OR "severe sepsis" [tiab] OR pyemia [tiab] OR pyemias [tiab] OR pyaemia [tiab] OR pyaemias [tiab] OR pyohemia [tiab] OR pyohemias [tiab]
**#3**	"Shock, Septic" [Mesh] OR "septic shock" [tiab] OR "endotoxic shock" [tiab] OR "endotoxin shock" [tiab] OR "endotoxin shocks" [tiab] OR "shock, endotoxic" [tiab] OR "shock, endotoxin" [tiab] OR "toxic shock" [tiab] OR "toxic shock syndrome" [tiab] OR "toxic shock syndromes" [tiab]
**#4**	"Shock" [Mesh] OR shock [tiab] OR "circulatory collapse" [tiab] OR "circulatory failure" [tiab] OR "hypovolemic shock" [tiab]
**#5**	#1 AND (#2 OR #3 OR #4)
Results	98

2. Cochrane Library

Search	Query
**#1**	[mh "anisodamine"] OR anisodamine:ti,ab,kw OR "anisodamine hydrobromide":ti,ab,kw OR racanisodamine:ti,ab,kw OR "6-hydroxyhyoscyamine":ti,ab,kw OR "654-2":ti,ab,kw OR "6542":ti,ab,kw OR "654 2":ti,ab,kw OR "654-1":ti,ab,kw OR "6541":ti,ab,kw OR "654 1":ti,ab,kw
**#2**	[mh "Sepsis"] OR sepsis:ti,ab,kw OR "bloodstream infection":ti,ab,kw OR "bloodstream infections":ti,ab,kw OR septicemia:ti,ab,kw OR septicemias:ti,ab,kw OR "blood poisoning":ti,ab,kw OR "blood poisonings":ti,ab,kw OR "severe sepsis":ti,ab,kw OR pyemia:ti,ab,kw OR pyemias:ti,ab,kw OR pyaemia:ti,ab,kw OR pyaemias:ti,ab,kw OR pyohemia:ti,ab,kw OR pyohemias:ti,ab,kw
**#3**	[mh "Shock, Septic"] OR "septic shock":ti,ab,kw OR "endotoxic shock":ti,ab,kw OR "endotoxin shock":ti,ab,kw OR "endotoxin shocks":ti,ab,kw OR "toxic shock":ti,ab,kw OR "toxic shock syndrome":ti,ab,kw OR "toxic shock syndromes":ti,ab,kw

**#4**	[mh "Shock"] OR shock:ti,ab,kw OR "circulatory collapse":ti,ab,kw OR "circulatory failure":ti,ab,kw OR "hypovolemic shock":ti,ab,kw
**#5**	#1 AND (#2 OR #3 OR #4)
Results	14

3. Web of Science

Search	Query
**#1**	TS=(anisodamine OR "anisodamine hydrobromide" OR racanisodamine OR "6-hydroxyhyoscyamine" OR "654-2" OR "6542" OR "654 2" OR "654-1" OR "6541" OR "654 1"))
**#2**	TS=(sepsis OR "bloodstream infection" OR "bloodstream infections" OR septicemia OR septicemias OR "blood poisoning" OR "blood poisonings" OR "severe sepsis" OR pyemia OR pyemias OR pyaemia OR pyaemias OR pyohemia OR pyohemias)
**#3**	TS=("septic shock" OR "endotoxic shock" OR "endotoxin shock" OR "endotoxin shocks" OR "toxic shock" OR "toxic shock syndrome" OR "toxic shock syndromes" OR shock OR "circulatory collapse" OR "circulatory failure" OR "hypovolemic shock")
**#4**	#1 AND (#2 OR #3)
Results	145

4. Embase

Search	Query
**#1**	'anisodamine'/exp OR anisodamine:ti,ab OR 'anisodamine hydrobromide':ti,ab OR racanisodamine:ti,ab OR '6 hydroxyhyoscyamine':ti,ab OR '6-hydroxyhyoscyamine':ti,ab OR '654-2':ti,ab OR '6542':ti,ab OR '654 2':ti,ab OR '654-1':ti,ab OR '6541':ti,ab OR '654 1':ti,ab
**#2**	'sepsis'/exp OR sepsis:ti,ab OR 'bloodstream infection':ti,ab OR 'bloodstream infections':ti,ab OR septicemia:ti,ab OR septicemias:ti,ab OR 'blood poisoning':ti,ab OR 'blood poisonings':ti,ab OR 'severe sepsis':ti,ab OR pyemia:ti,ab OR pyemias:ti,ab OR pyaemia:ti,ab OR pyaemias:ti,ab OR pyohemia:ti,ab OR pyohemias:ti,ab
**#3**	'septic shock'/exp OR 'septic shock':ti,ab OR 'shock, septic':ti,ab OR 'shock, endotoxic':ti,ab OR 'endotoxic shock':ti,ab OR 'endotoxin shock':ti,ab OR 'endotoxin shocks':ti,ab OR 'shock, endotoxin':ti,ab OR 'shock, toxic':ti,ab OR 'toxic shock':ti,ab OR 'toxic shock syndrome':ti,ab OR 'toxic shock syndromes':ti,ab

**#4**	'shock'/exp OR shock:ti,ab OR 'circulatory collapse':ti,ab OR 'circulatory failure':ti,ab OR 'hypovolemic shock':ti,ab
**#5**	#1 AND (#2 OR #3 OR #4)
Results	226

5. CNKI (中国知网)

Search	Query
**#1**	SU %= "脓毒症" OR SU %= "脓毒性休克" OR SU %= "休克" OR SU %= "感染性休克"
**#2**	SU %= "山莨菪碱" OR SU %= "氢溴酸山莨菪碱" OR SU %= "6542" OR SU %= "6541"
**#3**	#1 AND #2
Results	332

6. Wanfang Data (万方数据)

Search	Query
**#1**	主题:("脓毒症" OR "脓毒性休克" OR "休克" OR "感染性休克")
**#2**	主题:("山莨菪碱" OR "氢溴酸山莨菪碱" OR "6542" OR "6541")
**#3**	#1 AND #2
Results	481

7. VIP Database for Chinese Technical Periodicals (维普网)

Search	Query
**#1**	M=脓毒症 OR M=脓毒性休克 OR M=休克 OR M=感染性休克
**#2**	M=山莨菪碱 OR M=氢溴酸山莨菪碱 OR M=6542 OR M=6541

**#3**	#1 AND #2
Results	657

Field abbreviations used in Chinese databases:

CNKI: SU = Subject

Wanfang: 主题 = Subject

VIP: M = Subject

S2 Risk of Bias Assessment

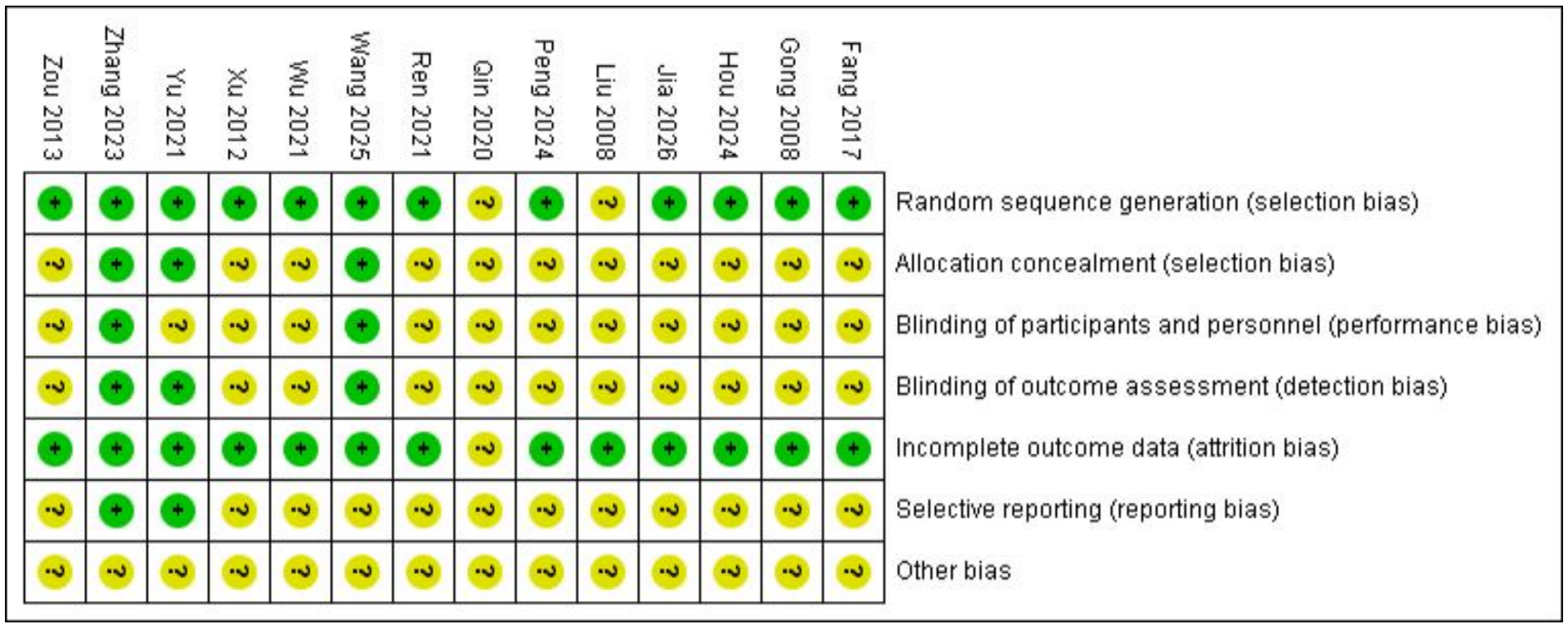

S3 Funnel Plots

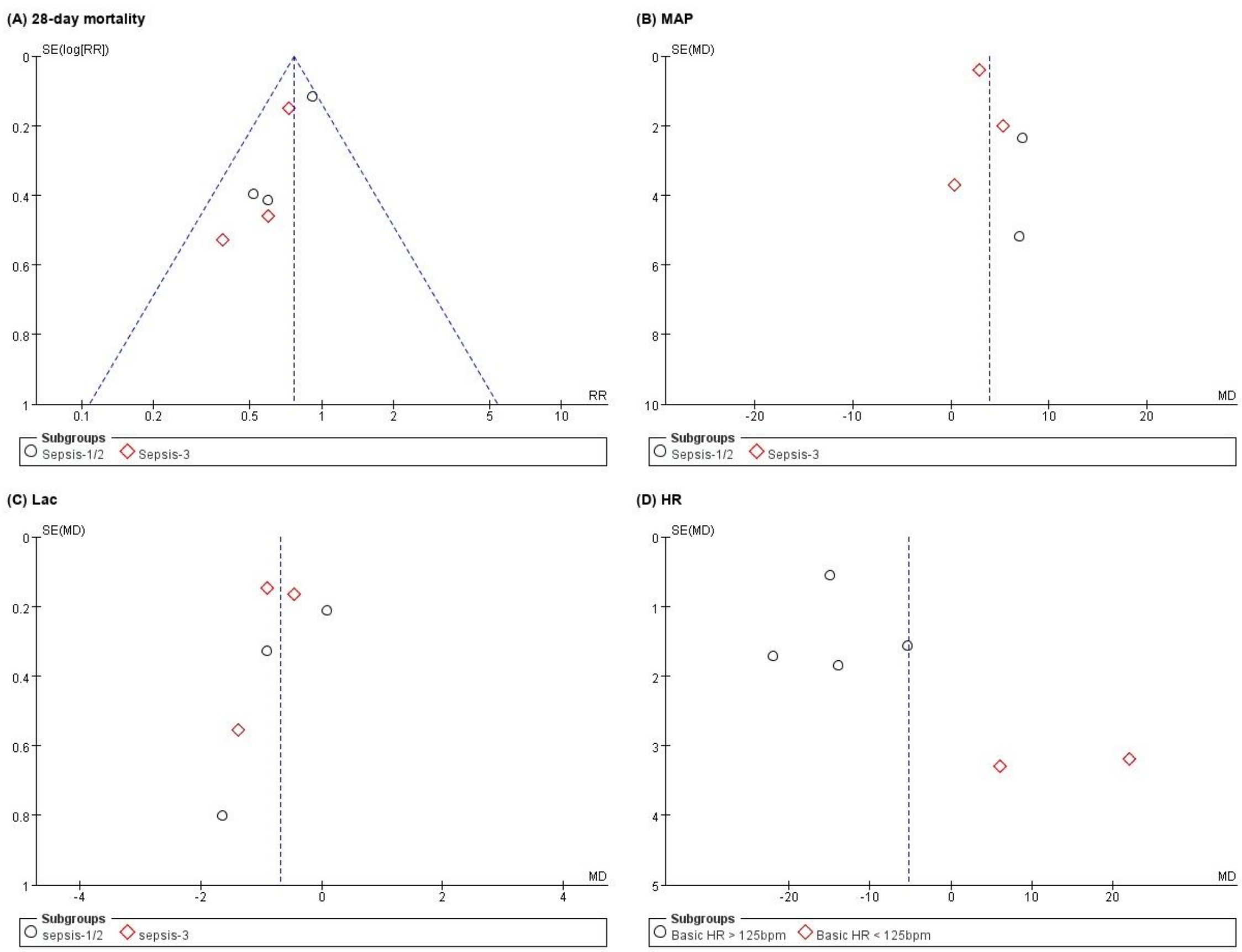

S4 Forest plot of MAP in anisodamine vs. control, stratified by dose

	Anisodamine			Control				Mean Difference
Study or Subgroup	Mean	SD	Total	Mean	SD	Total	Weight	IV, Random, 95% CI
**1.5.1 Dose >0.1 mg/(kg·h)**								
Gong 2008	77.5	14.1	15	70.5	14.3	15	3.9%	7.00 [-3.16, 17.16]
Qin 2020	95.93	13.77	30	95.66	15.41	32	7.1%	0.27 [-7.00, 7.54]
Wang 2025	80.66	10.59	55	75.41	10.32	55	19.1%	5.25 [1.34, 9.16]
**Subtotal (95% CI)**			**100**			**102**	**30.1%**	**4.43 [1.17, 7.69]**
Heterogeneity: Tau² = 0.00; Chi² = 1.67, df = 2 (P = 0.43); I² = 0%								
Test for overall effect: Z = 2.66 (P = 0.008)								
**1.5.2 Dose <0.1 mg/(kg·h)**								
Wu 2021	83	3.5	80	80.2	1.2	80	55.0%	2.80 [1.99, 3.61]
Xu 2012	66.04	9.25	24	58.77	6.73	22	14.9%	7.27 [2.62, 11.92]
**Subtotal (95% CI)**			**104**			**102**	**69.9%**	**4.43 [0.21, 8.64]**
Heterogeneity: Tau² = 7.09; Chi² = 3.45, df = 1 (P = 0.06); I² = 71%								
Test for overall effect: Z = 2.06 (P = 0.04)								
**Total (95% CI)**			**204**			**204**	**100.0%**	**3.92 [1.85, 5.98]**
Heterogeneity: Tau² = 1.85; Chi² = 5.88, df = 4 (P = 0.21); I² = 32%								
Test for overall effect: Z = 3.71 (P = 0.0002)								
Test for subgroup differences: Chi² = 0.00, df = 1 (P = 1.00), I² = 0%								

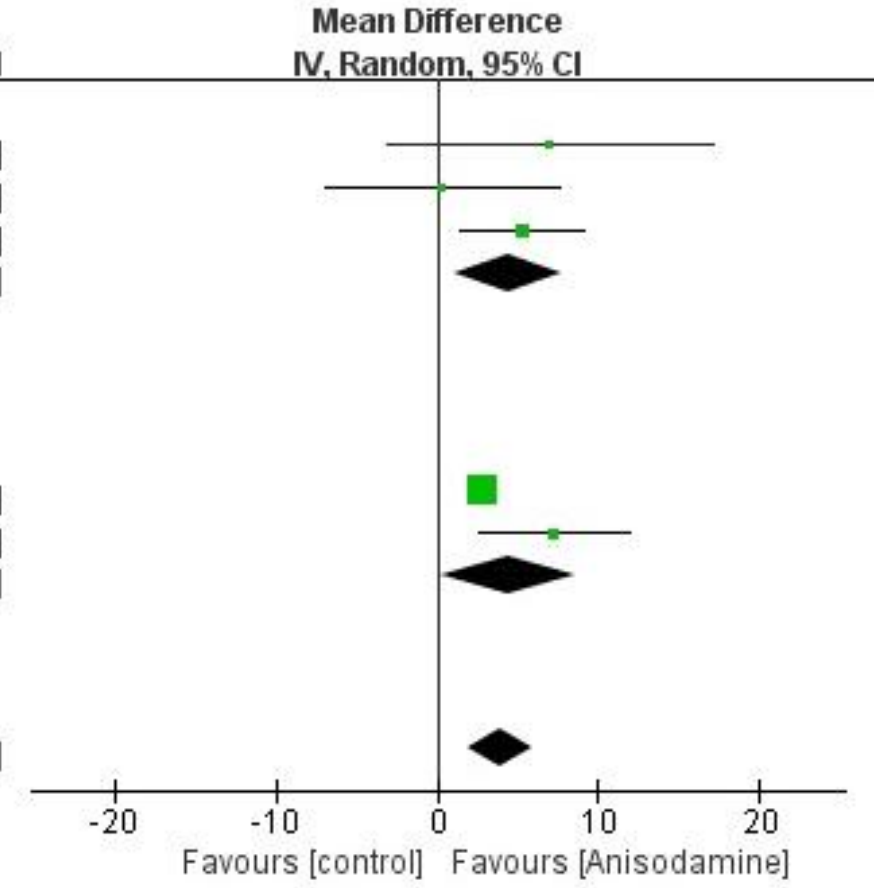

S5 Forest plot of HR in anisodamine vs. control, stratified by dose

	Anisodamine			Control				Mean Difference
Study or Subgroup	Mean	SD	Total	Mean	SD	Total	Weight	IV, Random, 95% CI
**1.14.1 Dose >0.1 mg/(kg·h)**								
Fang 2017	104	12	66	118	9	66	16.9%	-14.00 [-17.62, -10.38]
Gong 2008	119.2	10.1	15	97.2	7.1	15	15.9%	22.00 [15.75, 28.25]
Hou 2024	81	7	51	103	10	51	17.0%	-22.00 [-25.35, -18.65]
Jia 2026	88.63	5.06	131	103.62	3.81	131	17.4%	-14.99 [-16.07, -13.91]
Ren 2021	98.79	6.69	35	104.2	6.49	36	17.0%	-5.41 [-8.48, -2.34]
**Subtotal (95% CI)**			**298**			**299**	**84.2%**	**-7.32 [-15.98, 1.34]**
Heterogeneity: Tau² = 93.83; Chi² = 182.52, df = 4 (P < 0.00001); I² = 98%								
Test for overall effect: Z = 1.66 (P = 0.10)								
**1.14.2 Dose <0.1 mg/(kg·h)**								
Xu 2012	88.42	13.01	24	82.41	9.12	22	15.8%	6.01 [-0.44, 12.46]
**Subtotal (95% CI)**			**24**			**22**	**15.8%**	**6.01 [-0.44, 12.46]**
Heterogeneity: Not applicable								
Test for overall effect: Z = 1.83 (P = 0.07)								
**Total (95% CI)**			**322**			**321**	**100.0%**	**-5.18 [-13.53, 3.17]**
Heterogeneity: Tau² = 104.11; Chi² = 217.84, df = 5 (P < 0.00001); I² = 98%								
Test for overall effect: Z = 1.22 (P = 0.22)								
Test for subgroup differences: Chi² = 5.86, df = 1 (P = 0.02), I² = 82.9%								

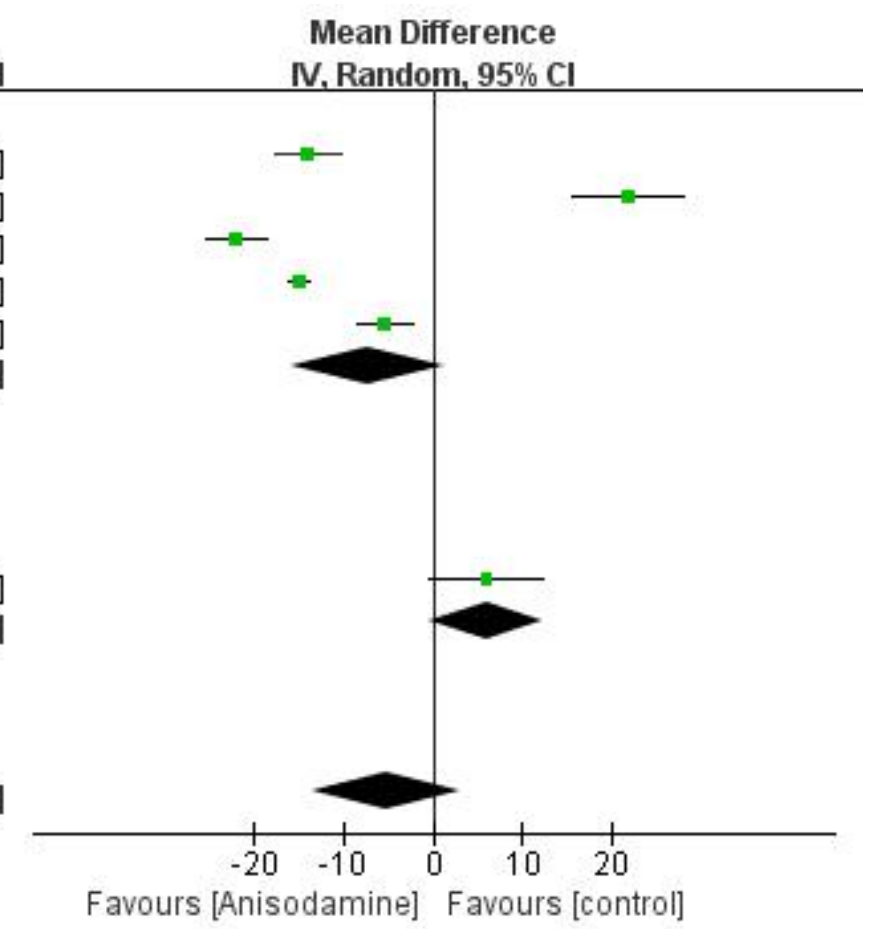

S6 Adverse reactions reported in included studies [n, n (%)]

Adverse reaction	Yu 2021 (T=181, C=174) T / C	Zhang 2023 (T=203, C=201) T / C	Wang 2025 (T=55, C=55) T / C	Jia 2026 (T=131, C=131) T / C
Tachycardia / Arrhythmia	0 / 0	2 (0.99) / 1 (0.50)	2 (3.64) / 2 (3.64)	—
Abdominal distension / Ileus	0 / 0	1 (0.49) / 0	—	—
Urinary retention	0 / 0	1 (0.49) / 0	1 (1.82) / 1 (1.82)	—
Blurred vision / Mydriasis	0 / 0	0 / 0	—	6 (4.58) / 0
Dry mouth	0 / 0	0 / 0	—	—
Facial flushing	0 / 0	0 / 0	2 (3.64) / 1 (1.82)	—
Local tissue edema	—	—	—	5 (3.82) / 16 (12.21)
Dizziness	—	—	—	1 (0.76) / 7 (5.34)
Headache	—	—	—	0 / 4 (3.05)
Nausea	—	—	—	3 (2.29) / 11 (8.40)
Vomiting	—	—	—	0 / 4 (3.05)
Consciousness disturbance	—	—	2 (3.64) / 1 (1.82)	—
Total	0 / 0	4 (1.97) / 1 (0.50)	7 (12.73) / 5 (9.09)	15 (11.45) / 42 (32.06)
Between-group comparison	Not reported	Not reported	$\chi^2 = 0.37$<br>$P = 0.541$	$\chi^2 = 16.346$<br>$P < 0.001$

T = anisodamine treatment group; C = control group.
"0" = the study pre-specified this adverse reaction but no events were observed. "—" = not reported in the study.
Yu 2021: Pre-specified monitoring included ileus, urinary retention, tachyarrhythmia (major); dry mouth, facial flushing, mild mydriasis, and blurred near vision (minor). No obvious anisodamine-related adverse reactions were reported in either group. Between-group P values were not reported.
Zhang 2023: Adverse reactions in the treatment group were mostly associated with high loading doses and high infusion rates; one case of transient tachycardia occurred in the control group. The between-group difference was not statistically significant (P>0.05, exact value not reported).
Wang 2025: The control group received conventional emergency treatment; the treatment group received anisodamine hydrobromide in addition.
Jia 2026: The control group received norepinephrine (NE) monotherapy; the treatment group received anisodamine plus NE. The incidence of NE-related adverse reactions was higher in the control group; these decreased with anisodamine co-treatment. Blurred vision, an anticholinergic effect of anisodamine, occurred only in the treatment group.

S7 Sensitivity analysis results

(A) Serum Lactate (Excluding Yu 2021)

	Anisodamine			Control				Mean Difference
**Study or Subgroup**	**Mean**	**SD**	**Total**	**Mean**	**SD**	**Total**	**Weight**	**IV, Random, 95% CI**
**1.9.1 sepsis-1/2**								
Liu 2008	3.13	1.85	21	4.78	3.16	21	3.8%	-1.65 [-3.22, -0.08]
Yu 2021	2.98	2.18	181	2.9	1.82	174	0.0%	0.08 [-0.34, 0.50]
Zou 2013	3.1	1.2	32	4	1.4	31	16.7%	-0.90 [-1.54, -0.26]
**Subtotal (95% CI)**			**53**			**52**	**20.6%**	**-1.01 [-1.60, -0.41]**
Heterogeneity: Tau² = 0.00; Chi² = 0.75, df = 1 (P = 0.39); I² = 0%								
Test for overall effect: Z = 3.32 (P = 0.0009)								
**1.9.2 sepsis-3**								
Qin 2020	2.26	1.35	30	3.65	2.8	32	7.4%	-1.39 [-2.47, -0.31]
Wu 2021	2.2	0.7	80	3.1	1.1	80	37.3%	-0.90 [-1.19, -0.61]
Zhang 2023	2.97	1.61	203	3.43	1.67	201	34.7%	-0.46 [-0.78, -0.14]
**Subtotal (95% CI)**			**313**			**313**	**79.4%**	**-0.77 [-1.18, -0.36]**
Heterogeneity: Tau² = 0.08; Chi² = 5.52, df = 2 (P = 0.06); I² = 64%								
Test for overall effect: Z = 3.66 (P = 0.0003)								
**Total (95% CI)**			**366**			**365**	**100.0%**	**-0.81 [-1.13, -0.49]**
Heterogeneity: Tau² = 0.05; Chi² = 7.02, df = 4 (P = 0.13); I² = 43%								
Test for overall effect: Z = 5.00 (P < 0.00001)								
Test for subgroup differences: Chi² = 0.43, df = 1 (P = 0.51), I² = 0%								

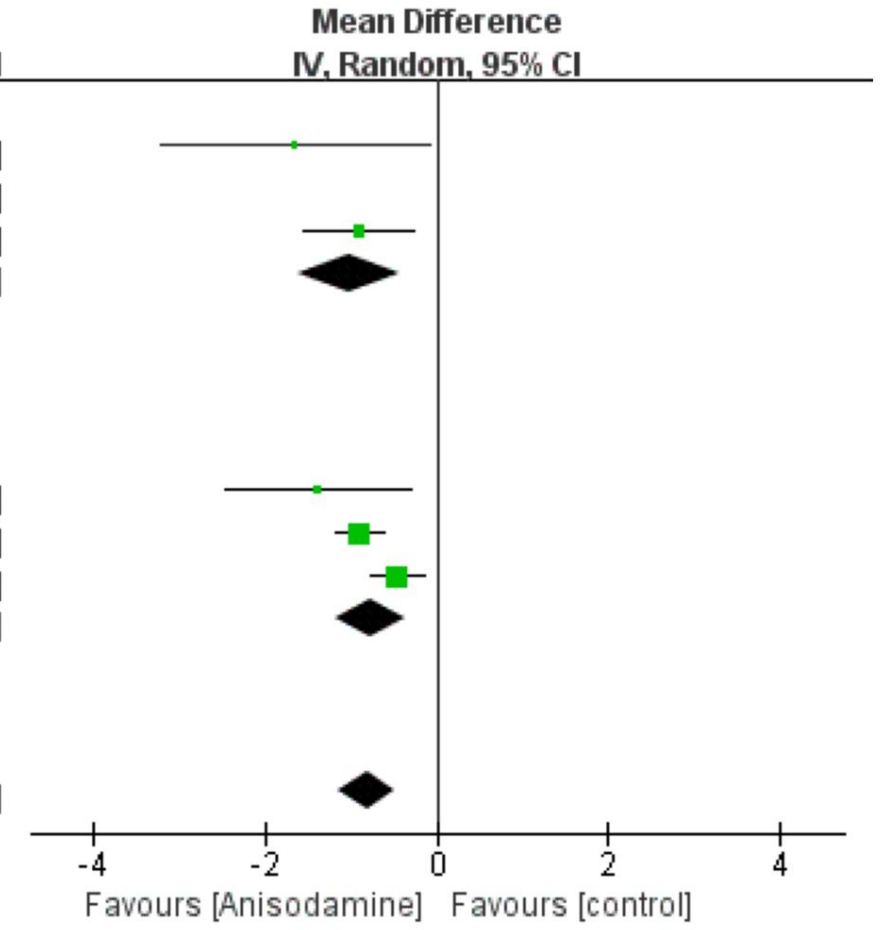

(B) SOFA Score (Excluding Wang 2025)

	Anisodamine			Control				Mean Difference
**Study or Subgroup**	**Mean**	**SD**	**Total**	**Mean**	**SD**	**Total**	**Weight**	**IV, Random, 95% CI**
Wang 2025	8.42	1.57	55	10.45	2.38	55	0.0%	-2.03 [-2.78, -1.28]
Yu 2021	4.66	2.97	181	5.17	3.7	174	59.3%	-0.51 [-1.21, 0.19]
Zhang 2023	5.49	5.25	203	6.56	5.35	201	27.1%	-1.07 [-2.10, -0.04]
Zou 2013	5.8	2.8	32	6.8	3.1	31	13.6%	-1.00 [-2.46, 0.46]
**Total (95% CI)**			**416**			**406**	**100.0%**	**-0.73 [-1.27, -0.19]**
Heterogeneity: Tau² = 0.00; Chi² = 0.93, df = 2 (P = 0.63); I² = 0%								
Test for overall effect: Z = 2.65 (P = 0.008)								

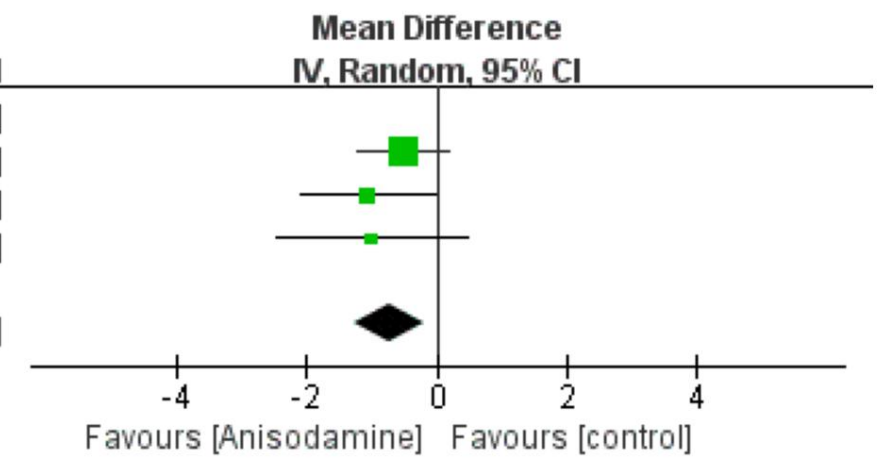

S8. Sensitivity Analysis of Outcomes After Removing Yu 2021

(A) 28-day mortality, stratified by diagnostic criteria

Study or Subgroup	Anisodamine Events	Anisodamine Total	Control Events	Control Total	Weight	Risk Ratio M-H, Random, 95% CI
**1.1.1 Sepsis-1/2**						
Liu 2008	6	21	10	21	9.1%	0.60 [0.27, 1.35]
Yu 2021	78	181	82	174	0.0%	0.91 [0.73, 1.15]
Zou 2013	7	32	13	31	9.9%	0.52 [0.24, 1.13]
**Subtotal (95% CI)**		**53**		**52**	**19.0%**	**0.56 [0.32, 0.98]**
Total events	13		23			
Heterogeneity: Tau² = 0.00; Chi² = 0.06, df = 1 (P = 0.81); I² = 0%						
Test for overall effect: Z = 2.04 (P = 0.04)						
**1.1.2 Sepsis-3**						
Peng 2024	6	36	10	36	7.4%	0.60 [0.24, 1.48]
Qin 2020	4	30	11	32	5.6%	0.39 [0.14, 1.09]
Zhang 2023	53	203	72	201	68.0%	0.73 [0.54, 0.98]
**Subtotal (95% CI)**		**269**		**269**	**81.0%**	**0.69 [0.52, 0.90]**
Total events	63		93			
Heterogeneity: Tau² = 0.00; Chi² = 1.43, df = 2 (P = 0.49); I² = 0%						
Test for overall effect: Z = 2.73 (P = 0.006)						
**Total (95% CI)**		**322**		**321**	**100.0%**	**0.66 [0.52, 0.84]**
Total events	76		116			
Heterogeneity: Tau² = 0.00; Chi² = 1.91, df = 4 (P = 0.75); I² = 0%						
Test for overall effect: Z = 3.34 (P = 0.0008)						
Test for subgroup differences: Chi² = 0.42, df = 1 (P = 0.52), I² = 0%						

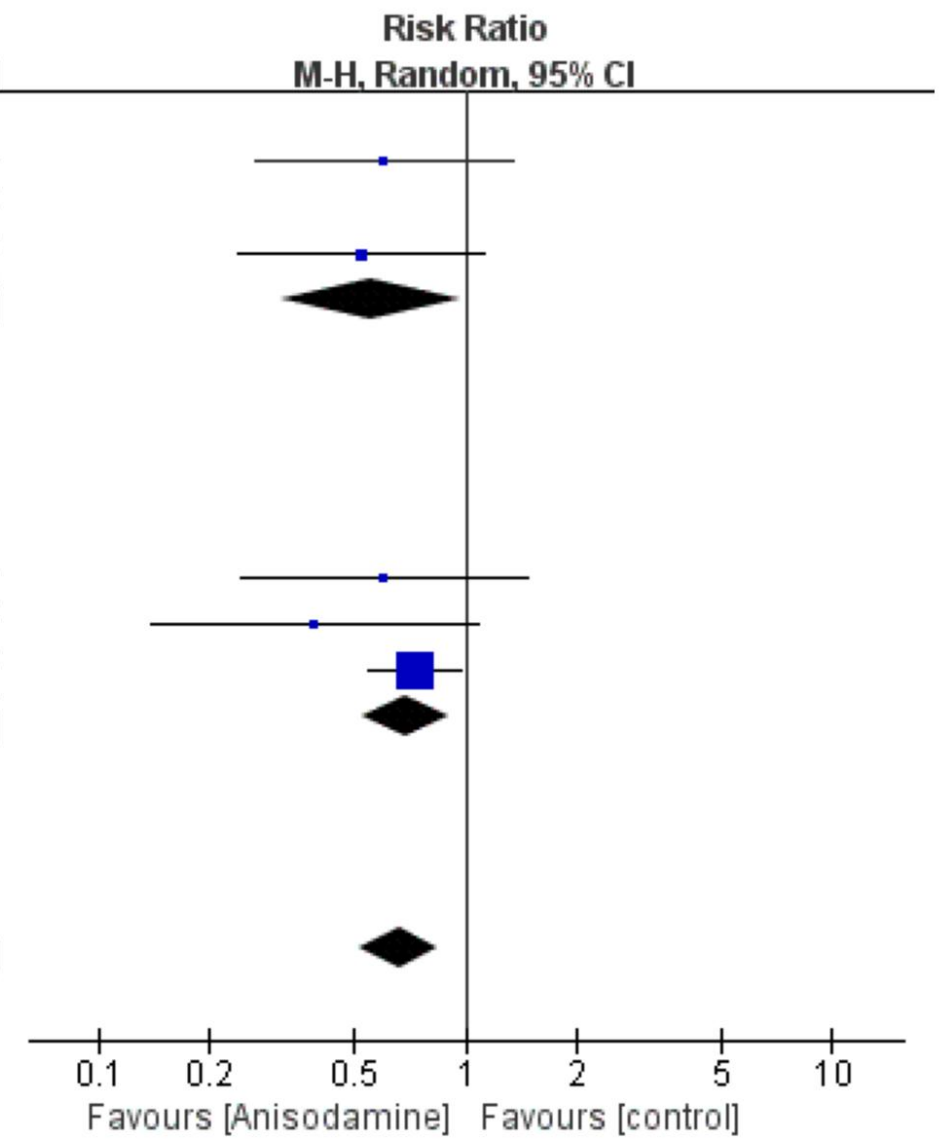

(B) 28-day mortality, stratified by risk of bias

Study or Subgroup	Anisodamine Events	Anisodamine Total	Control Events	Control Total	Weight	Risk Ratio M-H, Random, 95% CI
**1.2.1 Low risk of bias**						
Yu 2021	78	181	82	174	0.0%	0.91 [0.73, 1.15]
Zhang 2023	53	203	72	201	68.0%	0.73 [0.54, 0.98]
**Subtotal (95% CI)**		**203**		**201**	**68.0%**	**0.73 [0.54, 0.98]**
Total events	53		72			
Heterogeneity: Not applicable						
Test for overall effect: Z = 2.09 (P = 0.04)						
**1.2.2 Moderate/high risk of bias**						
Liu 2008	6	21	10	21	9.1%	0.60 [0.27, 1.35]
Peng 2024	6	36	10	36	7.4%	0.60 [0.24, 1.48]
Qin 2020	4	30	11	32	5.6%	0.39 [0.14, 1.09]
Zou 2013	7	32	13	31	9.9%	0.52 [0.24, 1.13]
**Subtotal (95% CI)**		**119**		**120**	**32.0%**	**0.53 [0.35, 0.82]**
Total events	23		44			
Heterogeneity: Tau² = 0.00; Chi² = 0.52, df = 3 (P = 0.91); I² = 0%						
Test for overall effect: Z = 2.86 (P = 0.004)						
**Total (95% CI)**		**322**		**321**	**100.0%**	**0.66 [0.52, 0.84]**
Total events	76		116			
Heterogeneity: Tau² = 0.00; Chi² = 1.91, df = 4 (P = 0.75); I² = 0%						
Test for overall effect: Z = 3.34 (P = 0.0008)						
Test for subgroup differences: Chi² = 1.39, df = 1 (P = 0.24), I² = 27.9%						

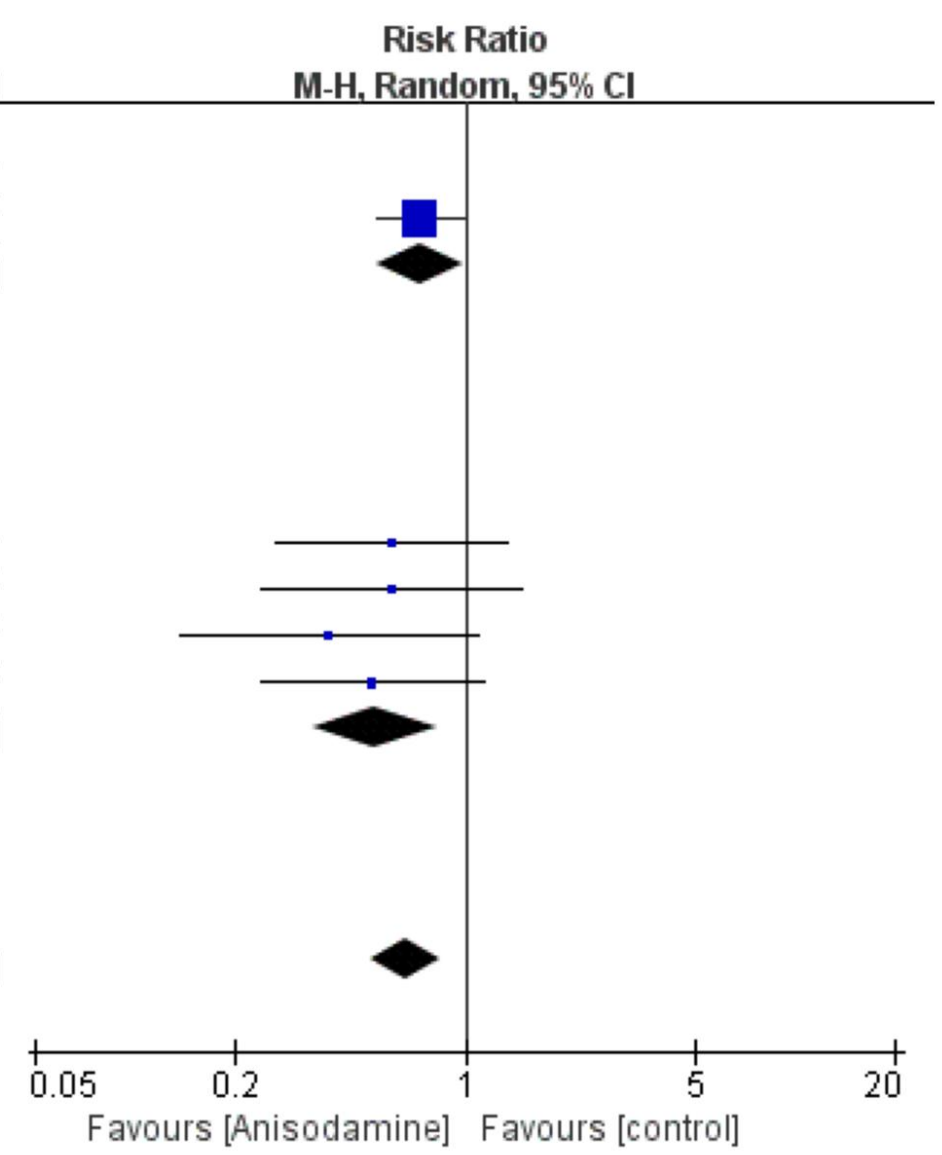

(C) Lac, stratified by diagnostic criteria

Study or Subgroup	Anisodamine Mean	SD	Total	Control Mean	SD	Total	Weight	Mean Difference IV, Random, 95% CI
**1.9.1 sepsis-1/2**								
Liu 2008	3.13	1.85	21	4.78	3.16	21	3.8%	-1.65 [-3.22, -0.08]
Yu 2021	2.98	2.18	181	2.9	1.82	174	0.0%	0.08 [-0.34, 0.50]
Zou 2013	3.1	1.2	32	4	1.4	31	16.7%	-0.90 [-1.54, -0.26]
**Subtotal (95% CI)**			**53**			**52**	**20.6%**	**-1.01 [-1.60, -0.41]**
Heterogeneity: Tau² = 0.00; Chi² = 0.75, df = 1 (P = 0.39); I² = 0%								
Test for overall effect: Z = 3.32 (P = 0.0009)								
**1.9.2 sepsis-3**								
Qin 2020	2.26	1.35	30	3.65	2.8	32	7.4%	-1.39 [-2.47, -0.31]
Wu 2021	2.2	0.7	80	3.1	1.1	80	37.3%	-0.90 [-1.19, -0.61]
Zhang 2023	2.97	1.61	203	3.43	1.67	201	34.7%	-0.46 [-0.78, -0.14]
**Subtotal (95% CI)**			**313**			**313**	**79.4%**	**-0.77 [-1.18, -0.36]**
Heterogeneity: Tau² = 0.08; Chi² = 5.52, df = 2 (P = 0.06); I² = 64%								
Test for overall effect: Z = 3.66 (P = 0.0003)								
**Total (95% CI)**			**366**			**365**	**100.0%**	**-0.81 [-1.13, -0.49]**
Heterogeneity: Tau² = 0.05; Chi² = 7.02, df = 4 (P = 0.13); I² = 43%								
Test for overall effect: Z = 5.00 (P < 0.00001)								
Test for subgroup differences: Chi² = 0.43, df = 1 (P = 0.51), I² = 0%								

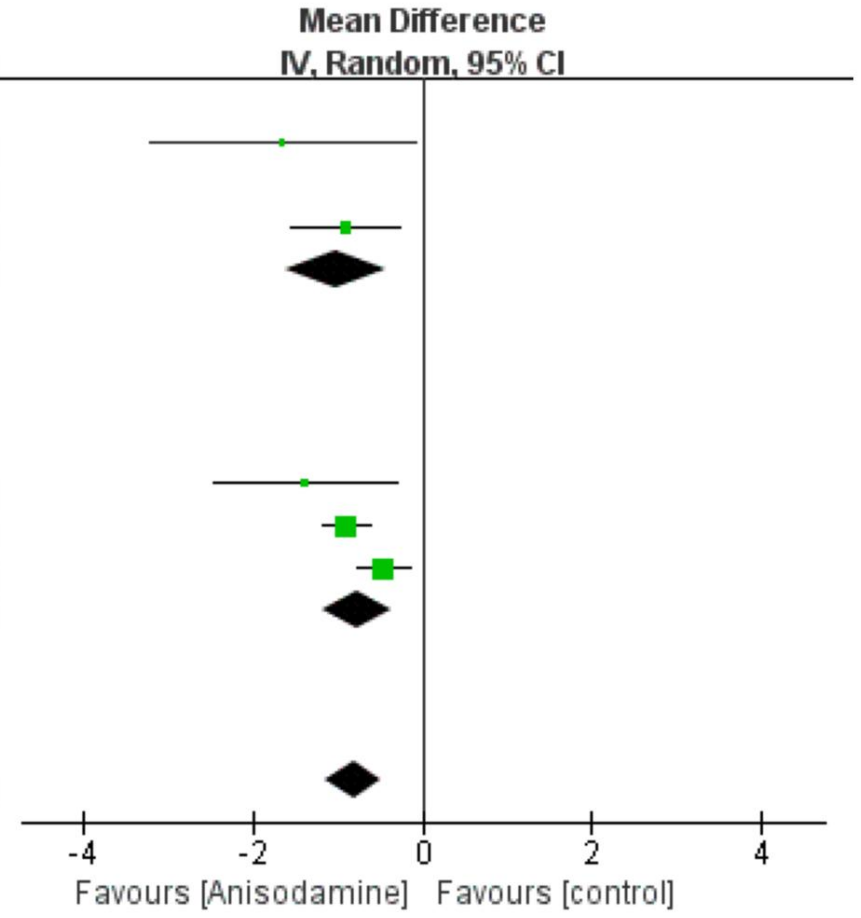

(D) SOFA Score

Study or Subgroup	Anisodamine Mean	SD	Total	Control Mean	SD	Total	Weight	Mean Difference IV, Random, 95% CI
Wang 2025	8.42	1.57	55	10.45	2.38	55	48.0%	-2.03 [-2.78, -1.28]
Yu 2021	4.66	2.97	181	5.17	3.7	174	0.0%	-0.51 [-1.21, 0.19]
Zhang 2023	5.49	5.25	203	6.56	5.35	201	32.6%	-1.07 [-2.10, -0.04]
Zou 2013	5.8	2.8	32	6.8	3.1	31	19.4%	-1.00 [-2.46, 0.46]
**Total (95% CI)**			**290**			**287**	**100.0%**	**-1.52 [-2.23, -0.80]**
Heterogeneity: Tau² = 0.13; Chi² = 2.91, df = 2 (P = 0.23); I² = 31%								
Test for overall effect: Z = 4.17 (P < 0.0001)								

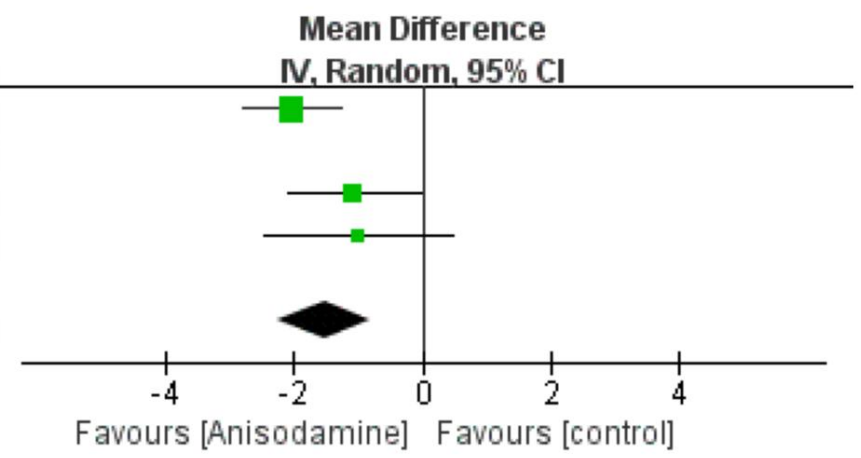

(E) Clinical Recovery Outcomes (Hospital LOs, ICU LOS, Vasopressor duration, and MV duration)

Study or Subgroup	Anisodamine Mean	SD	Total	Control Mean	SD	Total	Weight	Mean Difference IV, Random, 95% CI
**1.13.1 Hospital LOS**								
Peng 2024	12.17	5.04	36	17.72	8	36	47.3%	-5.55 [-8.64, -2.46]
Yu 2021	11.1	8.03	181	13.47	9.73	174	0.0%	-2.37 [-4.23, -0.51]
Zhang 2023	10	4.44	203	9	4.44	201	52.7%	1.00 [0.13, 1.87]
**Subtotal (95% CI)**			**239**			**237**	**100.0%**	**-2.10 [-8.51, 4.31]**
Heterogeneity: Tau² = 20.11; Chi² = 16.02, df = 1 (P < 0.0001); I² = 94%								
Test for overall effect: Z = 0.64 (P = 0.52)								
**1.13.2 ICU LOS**								
Peng 2024	8.77	3.7	36	13.72	8	36	28.2%	-4.95 [-7.83, -2.07]
Wang 2025	5.52	1.64	55	6.49	1.57	55	44.3%	-0.97 [-1.57, -0.37]
Yu 2021	6.26	4.75	181	6.77	5.8	174	0.0%	-0.51 [-1.62, 0.60]
Zou 2013	9.8	5	32	12.4	6.9	31	27.4%	-2.60 [-5.58, 0.38]
**Subtotal (95% CI)**			**123**			**122**	**100.0%**	**-2.54 [-5.02, -0.06]**
Heterogeneity: Tau² = 3.51; Chi² = 7.93, df = 2 (P = 0.02); I² = 75%								
Test for overall effect: Z = 2.01 (P = 0.04)								
**1.13.3 Vasopressor**								
Wang 2025	2.25	0.39	55	3.14	0.37	55	94.9%	-0.89 [-1.03, -0.75]
Yu 2021	6.06	11.41	181	7.41	14.33	174	0.0%	-1.35 [-4.05, 1.35]
Zhang 2023	2.83	3.7	203	3.83	4.07	201	3.3%	-1.00 [-1.76, -0.24]
Zou 2013	2.3	1.6	32	3.1	2.5	31	1.8%	-0.80 [-1.84, 0.24]
**Subtotal (95% CI)**			**290**			**287**	**100.0%**	**-0.89 [-1.03, -0.75]**
Heterogeneity: Tau² = 0.00; Chi² = 0.11, df = 2 (P = 0.95); I² = 0%								
Test for overall effect: Z = 12.63 (P < 0.00001)								
**1.13.4 MV duration**								
Wang 2025	4.16	1.25	55	5.33	1.47	55	96.9%	-1.17 [-1.68, -0.66]
Yu 2021	7.18	14.47	181	8.59	16.4	174	0.0%	-1.41 [-4.63, 1.81]
Zou 2013	8.1	5.1	32	9.9	6.3	31	3.1%	-1.80 [-4.64, 1.04]
**Subtotal (95% CI)**			**87**			**86**	**100.0%**	**-1.19 [-1.69, -0.69]**
Heterogeneity: Tau² = 0.00; Chi² = 0.18, df = 1 (P = 0.67); I² = 0%								
Test for overall effect: Z = 4.65 (P < 0.00001)								
Test for subgroup differences: Chi² = 3.04, df = 3 (P = 0.39), I² = 1.2%								

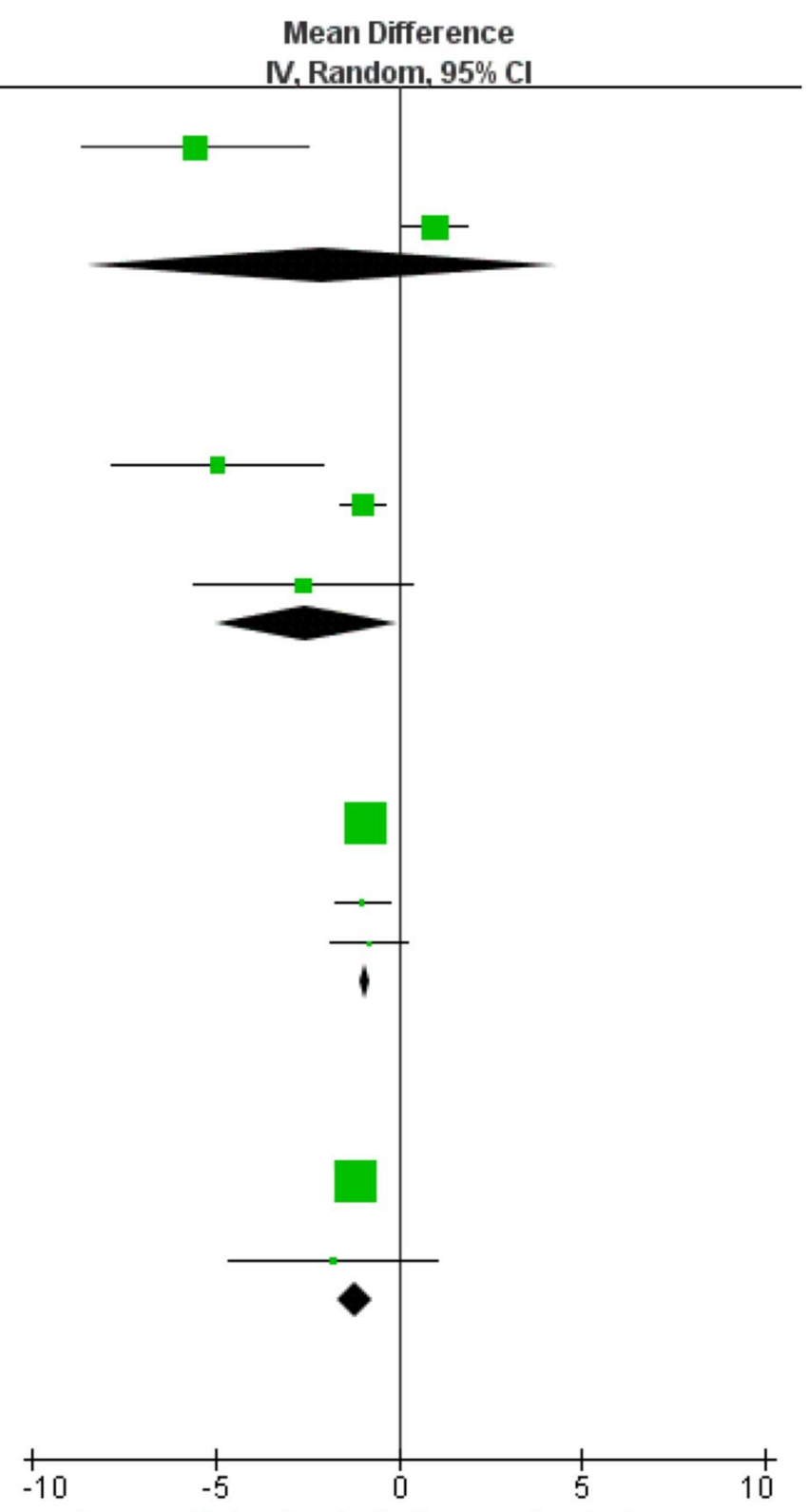

S9 Leave-one-out sensitivity analysis of vasopressor duration

Excluded study	MD (95% CI)	P for heterogeneity	I^2
None (all studies)	−0.89 (−1.03 to −0.76)	0.97	0%
Zou 2013	−0.89 (−1.03 to −0.76)	0.91	0%
Yu 2021	−0.89 (−1.03 to −0.75)	0.95	0%
Zhang 2023	−0.89 (−1.03 to −0.75)	0.93	0%
Wang 2025	−0.95 (−1.55 to −0.35)	0.91	0%

MD, mean difference; CI, confidence interval.

S10 Leave-one-out sensitivity analysis of MV duration

Excluded study	MD (95% CI)	P for heterogeneity	I^2
None (all studies)	−1.19 (−1.69 to −0.70)	0.90	0%
Zou 2013	−1.18 (−1.68 to −0.67)	0.89	0%
Yu 2021	−1.19 (−1.69 to −0.69)	0.67	0%
Wang 2025	−1.63 (−3.76 to 0.50)	0.86	0%

MV, mechanical ventilation; MD, mean difference; CI, confidence interval.

S11 GRADE Summary of Findings: Certainty of Evidence for Anisodamine as Adjuvant Therapy in Septic Shock

Outcome	No. of studies	Sample size (Tx/Ctrl)	Effect estimate (95% CI)	I^2	Risk of bias	Inconsistency	Indirectness	Imprecision	Publication bias	Certainty of evidence	Absolute effect (95% CI)
28-day mortality	6	998 (503/495)	RR 0.76 (0.62-0.93)	0.12	0.12	Serious[a]	Not serious	Not serious	Not serious	—(NE, n < 10)[φ]	⊕⊕⊕○ Moderate
MAP	5	408 (204/204)	MD 3.92 (1.85-5.98)	0.32	0.32	Serious[a]	Not serious	Not serious	Not serious	—(NE, n < 10)[φ]	⊕⊕⊕○ Moderate
SVRI	3	265 (131/134)	MD 74.78 (17.59-131.97)	0.53	0.53	Serious[a]	Serious[b]	Not serious	Not serious	—(NE, n < 10)[φ]	⊕⊕○○ Low
Cardiac Index (CI)	3	265 (131/134)	MD −0.02 (−0.64-0.61)	0.95	0.95	Serious[a]	Very serious[c]	Not serious	Serious[d]	—(NE, n < 10)[φ]	⊕○○○ Very Low
Lactate clearance rate	3	627 (315/312)	MD 16.27 (9.26-23.28)	0.87	0.87	Serious[a]	Not serious[e]	Not serious	Not serious	—(NE, n < 10)[φ]	⊕⊕⊕○ Moderate
Serum lactate	6	1086 (547/539)	MD −0.68 (−1.10-−0.26)	0.75	0.75	Serious[a]	Not serious[e]	Not serious	Not serious	—(NE, n < 10)[φ]	⊕⊕⊕○ Moderate
Oxygen delivery (DO_2)	3	305 (152/153)	MD 10.96 (−60.18-82.10)	0.97	0.97	Serious[a]	Very serious[c]	Not serious	Serious[d]	—(NE, n < 10)[φ]	⊕○○○ Very Low
Oxygen consumption (VO_2)	3	305 (152/153)	MD 23.10 (11.53-34.68)	0.72	0.72	Serious[a]	Serious[b]	Not serious	Not serious	—(NE, n < 10)[φ]	⊕⊕○○ Low
SOFA score	4	932 (471/461)	MD −1.17 (−1.95-−0.39)	0.65	0.65	Serious[a]	Not serious[e]	Not serious	Not serious	—(NE, n < 10)[φ]	⊕⊕⊕○ Moderate
APACHE-II score	2	182 (91/91)	MD −1.50 (−2.34-−0.65)	0	0	Serious[a]	Not serious	Not serious	Not serious	—(NE, n < 10)[φ]	⊕⊕⊕○ Moderate
Hospital LOS	3	831 (420/411)	MD −2.06 (−5.63-1.50)	0.92	0.92	Serious[a]	Very serious[c]	Not serious	Serious[d]	—(NE, n < 10)[φ]	⊕○○○ Very Low

Vasopressor duration	4	932 (471/461)	MD −0.89 (−1.03-−0.76)	0	0	Serious[a]	Not serious	Not serious	Not serious	—(NE, n < 10)[φ]	⊕⊕⊕○ Moderate
ICU LOS	4	600 (304/296)	MD −1.57 (−2.85-−0.29)	0.67	0.67	Serious[a]	Serious[b]	Not serious	Not serious	—(NE, n < 10)[φ]	⊕⊕○○ Low
Mechanical ventilation duration	3	528 (268/260)	MD −1.19 (−1.69-−0.70)	0	0	Serious[a]	Not serious	Not serious	Not serious	—(NE, n < 10)[φ]	⊕⊕⊕○ Moderate
Heart rate (safety)	6	643 (322/321)	MD −5.18 (−13.53-3.17)	0.98	0.98	Serious[a]	Very serious[c]	Not serious	Serious[d]	—(NE, n < 10)[φ]	⊕○○○ Very Low

[a] Risk of bias downgraded 1 level (serious): Most included RCTs lacked blinding and had inadequate allocation concealment. Subgroup analysis showed differential 28-day mortality effect estimates between studies with moderate/high vs. low risk of bias (RR 0.52 vs. 0.83), suggesting systematic influence of bias risk on effect estimates. All outcomes uniformly downgraded 1 level.

[b] Inconsistency downgraded 1 level (serious): I^2 = 53%-82%, substantial between-study heterogeneity in effect size that could not be fully explained by sensitivity/subgroup analyses.

[c] Inconsistency downgraded 2 levels (very serious): $I^2 \geq 92\%$, extremely high heterogeneity with significant between-study variation in effect direction and/or magnitude.

[d] Imprecision downgraded 1 level (serious): Pooled 95% CI crossed the null (RR = 1 or MD = 0), or the confidence interval was extremely wide; current evidence is insufficient to exclude null or harmful effects.

[e] Heterogeneity explained by sensitivity analysis, inconsistency not downgraded: Lactate clearance rate I^2 decreased to 27% after excluding Zou 2013; serum lactate I^2 decreased to 43% after excluding Yu 2021; SOFA I^2 decreased to 0% after excluding Wang 2025, with consistent direction of effect and robust conclusions. Sources of heterogeneity were identified; no downgrade for inconsistency, with reference to robust estimates after sensitivity analysis.

[φ] For remaining outcomes with < 10 studies, funnel plot test power was insufficient; marked as "—(NE, n < 10)".

Abbreviations: CI, confidence interval; RR, risk ratio; MD, mean difference; NNT, number needed to treat; MAP, mean arterial pressure; SVRI, systemic vascular resistance index; DO_2, oxygen delivery; VO_2, oxygen consumption; SOFA, Sequential Organ Failure Assessment; APACHE-II, Acute Physiology and Chronic Health Evaluation II; LOS, length of stay; ICU, intensive care unit; MV, mechanical ventilation; NE, not evaluated.
